# Bacterial population succession and adaptation affected by insecticide application and soil spraying history

**DOI:** 10.3389/fmicb.2014.00457

**Published:** 2014-08-29

**Authors:** Hideomi Itoh, Ronald Navarro, Kazutaka Takeshita, Kanako Tago, Masahito Hayatsu, Tomoyuki Hori, Yoshitomo Kikuchi

**Affiliations:** ^1^Bioproduction Research Institute, National Institute of Advanced Industrial Science and Technology (AIST)Sapporo, Japan; ^2^Research Institute for Environmental Management Technology, National Institute of Advanced Industrial Science and Technology (AIST)Tsukuba, Japan; ^3^Environmental Biofunction Division, National Institute for Agro-Environmental SciencesTsukuba, Japan

**Keywords:** fenitrothion, organophosphorus insecticide, soil microbes, deep sequencing, *Burkholderia*, methylotroph

## Abstract

Although microbial communities have varying degrees of exposure to environmental stresses such as chemical pollution, little is known on how these communities respond to environmental disturbances and how past disturbance history affects these community-level responses. To comprehensively understand the effect of organophosphorus insecticide application on microbiota in soils with or without insecticide-spraying history, we investigated the microbial succession in response to the addition of fenitrothion [*O*,*O*-dimethyl *O*-(3-methyl-*p*-nitrophenyl) phosphorothioate, abbreviated as MEP] by culture-dependent experiments and deep sequencing of 16S rRNA genes. Despite similar microbial composition at the initial stage, microbial response to MEP application was remarkably different between soils with and without MEP-spraying history. MEP-degrading microbes more rapidly increased in the soils with MEP-spraying history, suggesting that MEP-degrading bacteria might already exist at a certain level and could quickly respond to MEP re-treatment in the soil. Culture-dependent and -independent evaluations revealed that MEP-degrading *Burkholderia* bacteria are predominant in soils after MEP application, limited members of which might play a pivotal role in MEP-degradation in soils. Notably, deep sequencing also revealed that some methylotrophs dramatically increased after MEP application, strongly suggesting that these bacteria play a role in the consumption and removal of methanol, a harmful derivative from MEP-degradation, for better growth of MEP-degrading bacteria. This comprehensive study demonstrated the succession and adaptation processes of microbial communities under MEP application, which were critically affected by past experience of insecticide-spraying.

## Introduction

Biological communities are exposed to varying levels of environmental stresses or disturbances such as global warming, typhoon, drought, and bush fire (Phillips et al., 2009; O'Connell and Nyman, 2011; Peñuelas et al., 2013; Rota et al., 2014), and little is known about community succession and adaptation processes under such environmental stimuli. In addition to naturally occurring environmental disturbances, chemical insecticides have been developed and used worldwide to control agricultural, hygienic, and household pest insects. Although insecticides have revolutionized modern agriculture and sanitation in terms of pest management, abuse of usage sometimes causes serious problems including environmental pollution from its residues, health hazard, unexpected effects on non-targeted organisms, and evolution of insecticide-resistant insects (Whalon et al., 2008; Diez, 2010). Thus, insecticide-spraying is thought to be a man-made, yet strong, environmental stress on natural organisms.

Organophosphorus insecticide, a general name referring to insecticides containing a phosphoester bond, is one of the most widely used insecticides. It includes a number of commercially available chemicals such as diazinon, malathion, and dichlorvos (Singh and Walker, 2006; Singh, 2009). The chemical compounds found in organophosphorus insecticide show high mammalian toxicity as well as insecticidal activity by inhibiting acetylcholinesterase (AchE), causing overstimulation of the cholinergic synapses by the overaccumulation of acetylcholine (da Silva et al., 2013). Fenitrothion [*O*,*O*-dimethyl *O*-(3-methyl-*p*-nitrophenyl) phosphorothionate, abbreviated as MEP] is one of the organophosphorus compounds commonly used because of its broad-spectrum activity and less mammal toxicity. In natural fields, MEP can be degraded photochemically, but is mainly degraded by soil bacteria. Biodegradation pathways of MEP were first investigated in forest soils using C^14^-labeled MEP (Spillner et al., 1979) and are currently described in the International Programme on Chemical Safety IPCS (1992). In microorganisms, MEP is hydrolyzed to 3-methyl-4-nitrophenol and then finally decomposed to CO_2_ through a phenol ring cleavage pathway (IPCS, 1992). Previous culture-dependent studies reported that members of the genera *Burkholderia*, *Pseudomonas*, *Cupriavidus*, *Corynebacterium*, *Arthrobacter*, and *Sphingomonas* have been identified as MEP-degraders, some of which use the compound as a sole carbon source (Tago et al., 2006; Zhang et al., 2006; Kim et al., 2009). Considering that only a tiny fraction of environmental bacteria is culturable (Wilson and Piel, 2013), culture-independent as well as culture-dependent approaches are pivotal to comprehensively understand microbial dynamics in soils after spraying with MEP, as previously mentioned (Jacobsen and Hjelmsø, 2014).

Recent advances in sequence technologies, the so-called next generation sequencing (NGS), provide a faster and simpler alternative to previously established techniques for the comprehensive investigation of microbial community structures (Moorthie et al., 2011). Fairly recent works using NGS include the evaluation of microbial community compositions of soils polluted with organochlorine pesticides including Dichlorodiphenyltrichloroethane (DDT), hexachlorocyclohexane (HCH), and 2-chloro-4-ethylamino-6-isopropylamino-s-triazine (ATZ) (Fang et al., 2014). Based on the metagenomic survey of functional genes, these studies have suggested that diverse bacterial species complement one another to degrade these organochlorides (Fang et al., 2014).

In crop fields, biodegradation of the insecticides is one of the pivotal factors affecting the recovery and sustainability of soil ecosystems. Furthermore, it has been reported that one or more previous applications of the same insecticides and/or other structurally-analogous insecticides frequently result to less persistence of the insecticide in the environment (Felsot, 1989). In soil environments where the same insecticide has been continuously sprayed, it has been hypothesized that insecticide-degrading microbes grow by assimilating the insecticide and are enriched in the soil, which cause excessively enhanced biodegradation of the sprayed insecticides (Arbeli and Fuentes, 2007). However, it remains unclear how microbial communities transit under and adapt to insecticide-spraying and how the history of insecticide-spraying affects these succession and adaptation processes of soil microbial communities.

By combining the culture-independent approach using the NGS technique with culture-dependent isolation of MEP-degrading bacteria, we investigated the detailed process of microbial succession when MEP was sprayed on field-collected soils with or without a history of MEP-spraying prior to collection. Among the collected soils, one has been sprayed with MEP at least for 4 years and the other has never been sprayed with MEP nor with other organophosphorus compounds. The NGS analysis combined with culture-dependent approach clearly demonstrates previously unseen dynamics of soil microbiota during MEP-spraying, highlighting the different responses of microbiota against the experimental MEP-spraying in the different types of soil, and identifying the key players of MEP-degradation in soil environments.

## Materials and methods

### Soils

We collected two andisol soils from agricultural fields located at the National Institute for Agro-Environmental Sciences (Tsukuba, Ibaraki, Japan; 36°0′N, 140°1′E): *soil S* (*S*prayed soil) was collected from a crop field that had been sprayed with MEP for at least 4 years [pH(H_2_O), 6.3; water content (w/w), 35.2%; total carbon, 5.0%; total nitrogen, <0.3%]; *soil N* (*N*aive soil) was collected from a field that had never been sprayed with MEP and other organophosphorus compounds [pH(H_2_O), 6.6; water content (w/w), 33.7%; total carbon, 4.4%; total nitrogen, <0.3%]. Characterization of these soil samples is summarized in Table S1. Each soil was passed through a 2 mm sieve to remove large organic litters like leaves and roots.

### Insecticide-spraying experiments

Experimental design of the insecticide-spraying test is shown in Figure S1. Approximately 150 g (dry weight) of each of the sieved soils were transferred into plastic pots (10 × 8 cm; opening diameter × depth). The potted soils were incubated at 25°C with weekly application of MEP solution (diluted by distilled water) at a specific loading of 50 mg kg^−1^-dry soil (Tago et al., 2006). For both soils S and N, duplicate runs (pot 1 and pot 2) were prepared and individually subjected to experiments and further analyses. Control treatments were conducted under the same conditions, but the addition of MEP solution was replaced by equal amount of distilled water only once a week. These pots were covered with a sheet of aluminum foil during incubation to prevent water evaporation and photodegradation of MEP. Soil samples from the surface layer up to a depth of 1 cm were collected 1 week after the final spraying, e.g., soil samples treated three times with MEP were collected 1 week after the 3rd MEP-spraying (see Figure S1), and then subjected to the various analyses described below.

### CFU counting of MEP-degrading bacteria

The total CFU (colony forming units) counts of MEP-degrading bacteria was measured by plating serial dilutions of soil samples [1% (w/w) diluted in distilled water] on 1.5% agar plates of MEP medium [0.08% MEP, 20 mM potassium phosphate (pH 7.0), 0.1% (NH_4_)_2_SO_4_, 0.02% NaCl, 0.01% MgSO_4_•7H_2_O, 0.05% CaCl_2_•2H_2_O, 0.0002% FeSO_4_•7H_2_O, 0.1% yeast extract] and incubated for 4 days at 25°C, as previously described (Tago et al., 2006). The CFU of MEP-degrading bacteria were determined by counting the colonies with a halo on the MEP plates. In the halo-forming assay, the bacteria that are capable of degrading MEP to 3-methyl-4-nitrophenol were detected.

### Isolation and identification of MEP-degrading bacteria

Isolated colonies were identified based on the partial sequences (c.a. 600 bp) of their 16S rRNA genes. During isolation, each of the colonies was picked with a sterile toothpick and then suspended in 35 μl TE buffer [10 mM Tris-HCl, 1 mM EDTA (pH 8.0)] in each well of a 96 well titer plate. The cells were subjected to heat shock treatment (98°C for 3 min) in order to extract the genomic DNA, which was used as template for PCR amplification. The PCR amplification of bacterial 16S rRNA gene was performed with the use of a KOD Fx Neo polymerase (TOYOBO, Tokyo, Japan), a universal primer set, 16SA1 [5′-AGAGTTTGATCMTGGCTCAG-3′] and 16SB1 [5′-TACGGYTACCTTGTTACGACTT-3′] (Fukatsu and Nikoh, 1998), and the template DNA extracted as described above. The temperature program for PCR is as follows: 94°C for 2 min, followed by 20 cycles of 98°C for 10 s, 55°C for 30 s, and 68°C for 90 s. The resulting 1.5 kb amplicons were used as templates of sequencing reaction conducted with a universal primer 357F [5′-CCTACGGGAGGCAGCAG-3′] (Muyzer et al., 1993). Taxonomic assignment of the resulting sequences was double checked by the RDP multiclassifier ver. 1.1 (Wang et al., 2007) and BLASTN-search (http://ncbi.nlm.nih.gov/blast/) against GreenGene database (DeSantis et al., 2006).

### Deep sequencing

DNA was extracted from soil S and soil N samples before treatment (S0, N0), after the 2nd (S2) and 3rd (S3, N3) MEP-treatments, and after the 3rd control treatment (S3C, N3C) by using a slightly modified published protocol (Noll et al., 2005). Briefly, 0.5 g wet soil was combined with 0.08 g skim milk, 700 μl extraction buffer [50 mM Tris-HCl (pH 8.0), 1.7% Polyvinylpyrrolidone, 20 mM MgCl_2_], and 0.5 ml zirconia/silica beads (0.1 mm diameter, Biospec, OK, USA), and blended in a Multi-Beads Shocker (Yasui Kikai, Osaka, Japan) at 2000 rpm for 60 s. Crude extracts were purified by phenol/chloroform/isoamyl alcohol (25:24:1) treatment followed by isopropanol precipitation. Contaminating RNA was digested with Ribonuclease (Nippon Gene, Toyama, Japan). The prepared DNA was subjected to PCR amplification of 16S rRNA gene for deep sequencing. The variable region (V4) of bacterial 16S rRNA gene was amplified using universal primers 515F [5′-GTGCCAGCMGCCGCGGTAA-3′] and 806R [5′-GGACTACHVGGGTWTCTAAT-3′] (Caporaso et al., 2012). The PCR reaction mixture comprised of 50 μM each dNTP, 0.4 μM 515F with Illumina P5 sequences, 0.4 μM 806R with 6-bases indexes and Illumina P7 sequences (Caporaso et al., 2012) (Illumina, San Diego, CA, USA), Q5 High-Fidelity DNA polymerase with Q5 reaction buffer (New England BioLabs, Ipswich, MA, USA) and the extracted soil DNA template. The PCR conditions were as follows: initial denaturation at 98°C for 90 s, followed by 25 cycles of 98°C for 10 s, 54°C for 30 s, 72°C for 30 s, and a final extension at 72°C for 2 min. The PCR amplicons were purified as described previously (Itoh et al., 2014). DNA libraries containing all tagged-amplicons and internal control phiX were generated for paired-end sequencing by the MiSeq sequencer using MiSeq Reagent kit v2 (Illumina, San Diego, CA, USA) according to the manufacturer's instruction.

### Quantitative PCR

Quantitative PCR (qPCR) of bacterial 16S rRNA gene was performed to amplify bacterial 16S rRNA genes using a Power SYBR Green PCR Master Mix (Applied Biosystems, Foster City, CA, USA) and the LightCycler 96 System (Roche Applied Science, Indianapolis, IN, USA). The reaction mixture was comprised of 2× SYBR Green PCR Master Mix, 0.2 μM 515F and 806R primer pairs (Caporaso et al., 2012), 0.5 μg/μl BSA, and soil DNA as a template. The PCR conditions were as follows: initial denaturation at 95°C for 10 min, followed by 45 cycles of 95°C for 30 s, 57°C for 30 s, and 72°C for 30 s. The amount of bacterial 16S rRNA gene copies was calculated on the basis of the standard curve constructed using a dilution series of the target PCR product of *Burkholderia* sp. SFA1 (DDBJ accession no. AB232333).

### Data analysis

Internal control phiX sequences and low-quality sequences were removed, and paired sequences were joined as described previously (Itoh et al., 2014). Chimeric sequences were removed using uchime algorithm on Mothur program ver. 1.29.2 (Schloss et al., 2009) through the alignment with Greengene database (DeSantis et al., 2006). The resulting sequences were subjected to taxonomic assignment by using RDP multiclassifier ver. 1.1 (Wang et al., 2007) with an 80% confidence threshold. Operational taxonomic units (OTU) based analyses, including estimation of diversity indices and Principal Coordinate Analysis (PCoA) based on OTUs with 3% differences, were performed by using the macqiime ver. 1.6.0 (Caporaso et al., 2010). Phylogenetic tree was constructed by the neighbor-joining method with the bootstrap test (1000 replicates) under MEGA ver. 4.0.2 (Tamura et al., 2007). Analysis of variance (ANOVA) was performed using R software ver. 3.0.1 (R Development Core Team, 2008) to analyze differences in qPCR data among samples.

### Nucleotide sequence accession number

The nucleotide sequences reported in this study were deposited in the DDBJ/Genbank/EBI databases under the accession numbers: AB904935–AB905196 (16S rRNA gene sequences of isolates, Table S2) and the MG-RAST database (http://metagenomics.anl.gov/, Meyer et al., 2008) as a “Microbial succession under MEP-spraying in 2012” project under the ID: 4562358.3–4562369.3 (deep sequencing, Table 1).

**Table 1:** **Summary of deep sequencing and qPCR**.

**Soil**	**No. of spraying**	**Pot No.**	**Libarary ID**	**No. of sequences^b^**	**No. of OTU^c^_0.03_**	**Cx^d^**	**Diversity indices^e^**			**No. of copies^f^**
							**chao1**	**shannon**	**1/simpson**	
S	0	1	S0p1	13,202	1,206	0.97	1,944 ± 107	7.98 ± 0.04	65 ± 2.7	3.6 ± 0.1 × 10^8^
S	2	1	S2p1	13,346	796	0.99	1,188 ± 103	5.9 ± 0.03	15 ± 0.3	4.5 ± 0.3 × 10^8^
S	3	1	S3p1	14,600	575	0.99	813 ± 79	3.86 ± 0.05	4.5 ± 0.1	4.7 ± 0.2 × 10^8^
S	0	2	S0p2	6,218	1,062	0.93	2,446 ± 78	8.62 ± 0.01	109 ± 0.7	4.6 ± 0.2 × 10^8^
S	2	2	S2p2	10,223	1,154	0.94	2,324 ± 116	7.72 ± 0.03	42 ± 0.8	4.0 ± 0.6 × 10^8^
S	3	2	S3p2	8,213	897	0.96	1,731 ± 100	6.43 ± 0.03	13 ± 0.3	4.4 ± 0.5 × 10^8^
S	0^a^	control	S3C	17,933	1,533	0.94	2,260 ± 123	8.6 ± 0.04	124 ± 2.8	4.8 ± 1.2 × 10^8^
N	0	1	N0p1	13,540	1,500	0.85	3,268 ± 190	9.07 ± 0.04	138 ± 5.8	5.5 ± 1.8 × 10^8^
N	3	1	N3p1	10,171	1,028	0.95	1,919 ± 114	7.3 ± 0.03	36 ± 0.8	6.3 ± 0.9 × 10^8^
N	0	2	N0p2	15,054	1,747	0.87	3,655 ± 180	9.1 ± 0.02	151 ± 3.4	6.3 ± 0.2 × 10^8^
N	3	2	N3p2	34,865	1,970	0.90	2,977 ± 187	7.78 ± 0.04	33 ± 1.2	6.8 ± 0.5 × 10^8^
N	0^a^	control	N3C	72,066	2,459	0.90	3,470 ± 139	9.06 ± 0.04	149 ± 9.3	8.6 ± 1.0 × 10^8^

a Control pots received distilled water (DW) once a week: soils were collected 1 week after 3rd DW-spraying.

b Number of sequences after removal of low-quality, chimeric, and archaeal sequences.

c Clustered at the 3% distance level.

d Calculated from the equation, Cx = 1–(n/N), where “n” is the number of OTUs composed of only one sequence (singleton) and N is the total number of sequences.

e Each index was calculated based on the same amount of sequences (5174) sub-sampled from original libraries (Mean ± SDs of 10 time sub-samplings is shown).

f Copy numbers (copies g^−1^-dry soil) of 16S rRNA genes estimated by qPCR (Mean ± SDs of three time measurements of the same DNA sample is shown). Analysis of variance (ANOVA) was performed using R software ver. 3.0.1 (R Development Core Team, 2008), showing the copy numbers were not significantly different between samples that were originated from the same field soil.

## Results

### Population dynamics of MEP-degrading bacteria in MEP-sprayed soils

**Figure 1:**
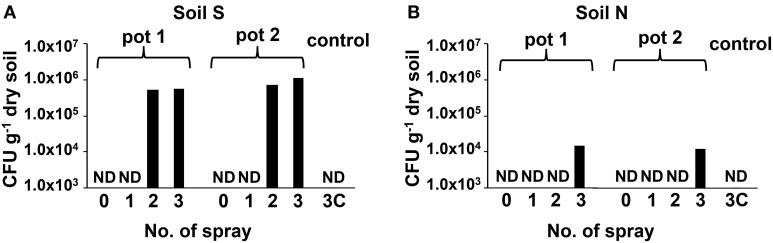
**Transition of CFU of MEP-degrading bacteria in MEP-sprayed soils.** CFU of MEP-degrading bacteria in soil S **(A)** and soil N **(B)** were counted by using MEP medium plates. Results of replicated experiments (pot 1 and pot 2) are shown. ND, not detected (<1.0 × 10^3^ cfu g^−1^-dry soil).

Abundance changes of MEP-degrading strains by a culture-dependent method were investigated using two types of soils with different MEP-spraying histories: MEP-sprayed “soil S” and MEP-naive “soil N” (Table S1). Although the CFU of MEP-degrading bacteria were below the detectable level (<10^3^ cfu g^−1^ dry soil) even 1 week after the insecticide application, they became detectable after the 2nd and 3rd treatments in soil S and soil N, respectively, (Figure 1). In the control, with only the addition of distilled water, no MEP-degraders were detected even after the 3rd treatment (Figure 1). These results indicate that the repeated spraying of MEP acts as a strong selective pressure on soil microbiota, confirming our findings from previous studies (Tago et al., 2006; Kikuchi et al., 2012). It has also been reported that some bacteria are capable of utilizing MEP as a sole carbon source, fostering their population increase (Hayatsu et al., 2000; Tago et al., 2006; Zhang et al., 2006; Kim et al., 2009), which likely explains the above-observed population dynamics of MEP-degrading strains in soil environments.

### Isolation and identification of MEP-degrading bacteria

**Figure 2:**
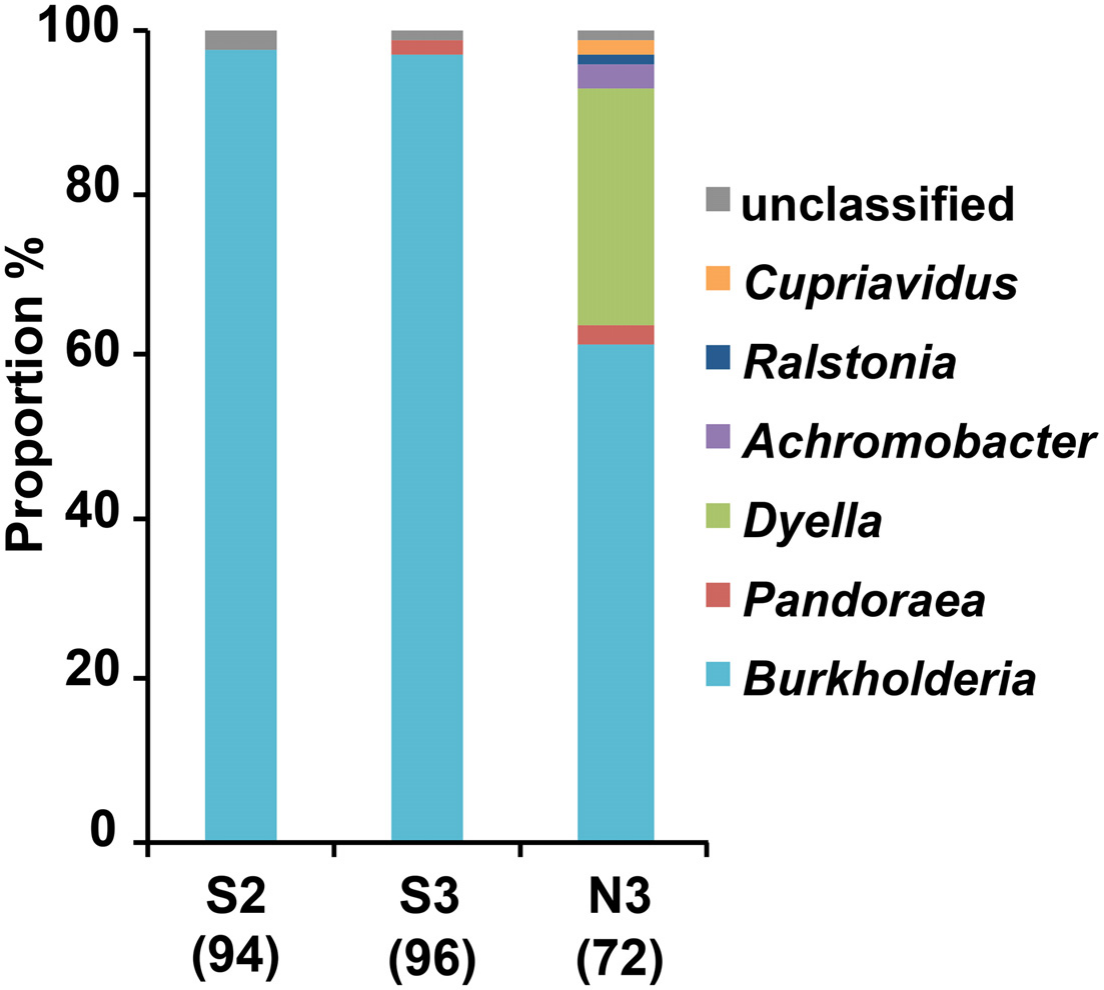
**Genus-level compositions of MEP-degrading bacteria isolated from MEP-sprayed soils.** MEP-degrading isolates from soil S after two times and three times MEP spraying (S2 and S3, respectively) and from soil N after three times MEP spraying (N3) were identified by partial (c.a. 600 bp) sequencing of 16S rRNA gene. Numbers in parentheses indicate total numbers of inspected MEP-degrading strains that were randomly selected from 2 pots in each soil (i.e., results of pots 1 and 2 are merged here). Sequences were subjected to taxonomic assignment by using RDP multiclassifier with a threshold level of 80%.

From the soil S after the 2nd and 3rd MEP-treatments (S2 and S3), and soil N after the 3rd MEP-treatment (N3), colonies of the MEP-degrading bacteria were isolated and identified based on the partial sequences of 16S rRNA gene. The sequence analyses demonstrated that the majority belonged to the betaproteobacterial genus *Burkholderia*. Specifically, they occupied around 97.9, 96.9, and 61.1% of the total sequenced-bacteria isolated from the S2, S3, and N3 soils, respectively, (Figure 2; Table S2). Notably, in contrast to the predominance of *Burkholderia* strains in the S2 and S3 samples, MEP-degraders from the N3 sample were more phylogenetically diverse with the presence of strains from the genera *Dyella*, *Ralstonia*, *Pandoraea*, *Achromobacter*, and *Cupriavidus*.

### Succession of microbiota in MEP-sprayed soils as revealed by deep sequencing

For comprehensive understanding of the temporal dynamics of microbiota in the MEP-sprayed soils, we performed deep sequencing of bacterial 16S rRNA genes from both soils before treatment (S0 and N0), after the 2nd (S2) and 3rd (S3 and N3) MEP-treatments, and after the 3rd control-treatment (S3C and N3C). The sequencing of PCR amplicons of partial 16S rRNA genes produced >2 × 10^5^ sequences in total (Table 1). The qPCR data showed that the amount of bacterial 16S rRNA genes was stable at around 4 × 10^8^ and 6 × 10^8^ g^−1^-dry soil in soil S and soil N, respectively, during the treatments (Table 1). Meanwhile, in both soils S and N, diversity indices, chao1, Shannon, and reciprocal simpson decreased after MEP-spraying (Table 1), suggesting that MEP treatment caused the decrease of soil microbial diversity.

Similarities among the sequence libraries of each soil sample were analyzed by PCoA based on weighted unifrac distance (Figure S2). The results suggest that the bacterial compositions of S2, S3, and N3 were distinct from those of S0 and N0 samples, although the shift direction was different between the soil S and soil N. The controls, S3C and N3C, were closely related to the soils before treatments, S0 and N0. These results also clearly demonstrate that MEP-spraying dramatically altered the community structure of soil microbiota.

### Bacterial groups responding to MEP-spraying

**Figure 3:**
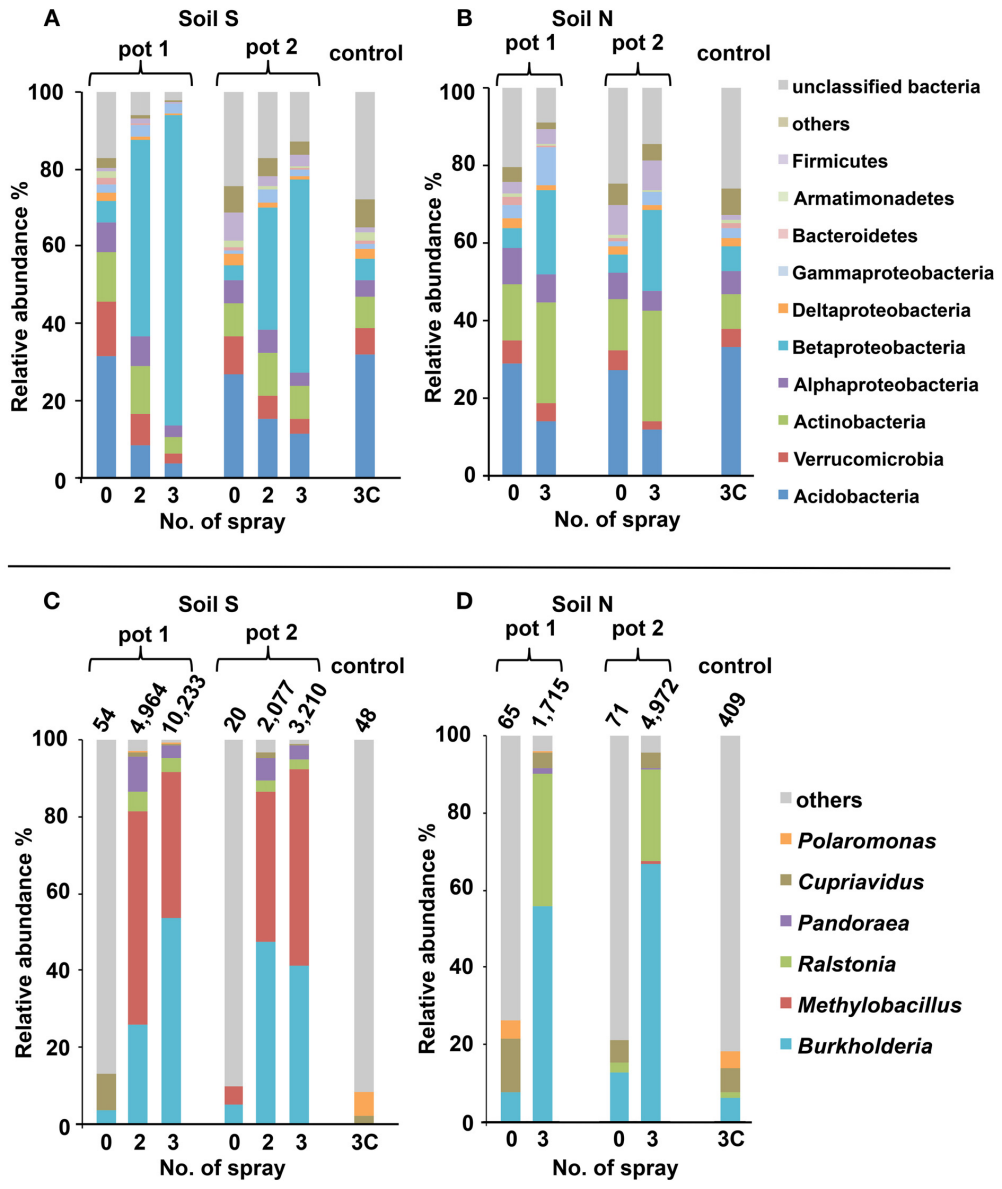
**Structural changes of microbial communities in MEP-sprayed soils.** Soil S **(A,C)** and soil N **(B,D)** were analyzed by deep sequencing of 16S rRNA gene. For each soil, results of replicated experiments (pot 1 and pot 2) are shown. Relative distributions of the sequences at **(A,B)** phylum-level (class-level in the *Proteobacteria*) and **(C,D)** genus-level within *Betaproteobacteria* are shown. In **(C,D)**, total numbers of betaproteobacterial sequences excluding those of unclassified genus are depicted on the bars. Sequences were subjected to taxonomic assignment by using RDP multiclassifier with a threshold level of 80%.

Figures 3A,B show the phylum-level (class-level in the *Proteobacteria*) distribution of sequences obtained from the deep sequence libraries. Among the diverse bacterial groups, *Betaproteobacteria* drastically increased after MEP-spraying in all samples from both soils S and N. Especially in soil S, the relative abundance of *Betaproteobacteria* was over 40% (80.4% in pot 1 and 49.8% in pot 2) after the 3rd MEP-treatment (Figure 3A). In the control samples (S3C and N3C), the bacterial composition appeared to be stable based from the initial states (S0 and N0), as also supported by the PCoA profile (Figure S2).

Within the increasing community of *Betaproteobacteria*, the genus *Burkholderia* was the most abundant, followed by *Ralstonia* and *Pandoraea* (Figures 3C,D). Before MEP treatment, few *Burkholderia* sequences were detected. However, after the 3rd treatment, the frequencies of *Burkholderia* drastically increased up to 53.8% in soil S pot 1, 41.1% in soil S pot 2, 55.7% in soil N pot 1, and 66.9% in soil N pot 2.

**Figure 4:**
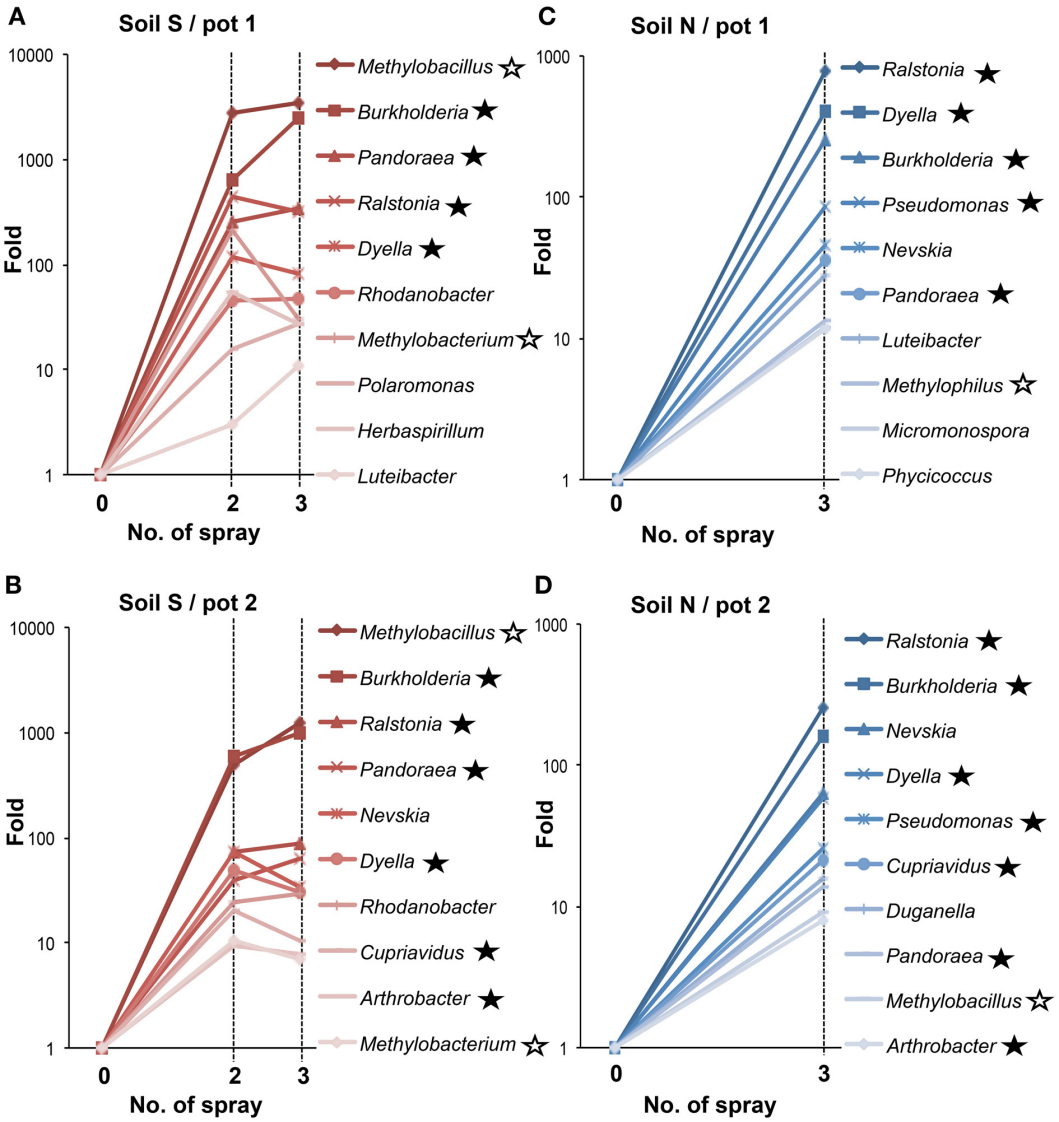
**Temporal changes of relative proportions of bacterial genera during MEP-spraying. (A,B)** Soil S; **(C,D)** soil N. Relative proportions of bacterial genera were estimated based on the read numbers of deep sequencing of 16S rRNA sequences. Closed asterisks indicate the genera, which have been reported to degrade MEP (Tago et al., 2006; Zhang et al., 2006; Kim et al., 2009), or are newly identified as MEP-degrading bacteria in this study (Figure 2). Open asterisks indicate methylotrophs. In addition to the relative increasing rates in MEP-sprayed soils, those in DW-sprayed soils (controls) are shown in text.

Figure 4 shows increase in the relative proportion of specific bacteria genera during the MEP-spraying experiments. It depicts the top 10 genera with the higher frequency after the 3rd treatment. From the list, the *Burkholderia*, *Cupriavidus*, *Pseudomonas*, and *Arthrobacter* are already well-known MEP-degrading strains (Tago et al., 2006; Zhang et al., 2006; Kim et al., 2009). Furthermore, the genera *Pandoraea*, *Ralstonia*, and *Dyella*, which were additionally identified earlier as MEP-degrading group (Figure 2), were also included in the top 10 genera that highly responded to MEP (Figure 4). In both soils S and N, the frequencies of *Burkholderia* and *Ralstonia* drastically increased in response to MEP-spraying at two to three orders of magnitude, suggesting that these genera might possess a strong MEP-assimilation ability to highly adapt to the biological niche resulting from MEP-sprayed soils. Other bacterial groups, such as *Rhodanobacter*, *Nevskia*, and *Methylobacillus*, were also included in the top 10 (Figure 4), although they were not detected through the culture-dependent approach (Figure 2). These strains might assimilate the intermediates of MEP degradation such as 3-methl-4-nitrophenol and p-nitrophenol (Hayatsu et al., 2000; Arora et al., 2013), and thus proliferate under the MEP treatment. The frequencies of these bacterial groups with high increasing ratios after MEP treatment were almost stable in the control set-ups in both of soils S and N (Fold: 0.2~3.4), emphasizing that these bacteria respond to MEP-spraying.

### Burkholderia strains responding to MEP-spraying

**Figure 5:**
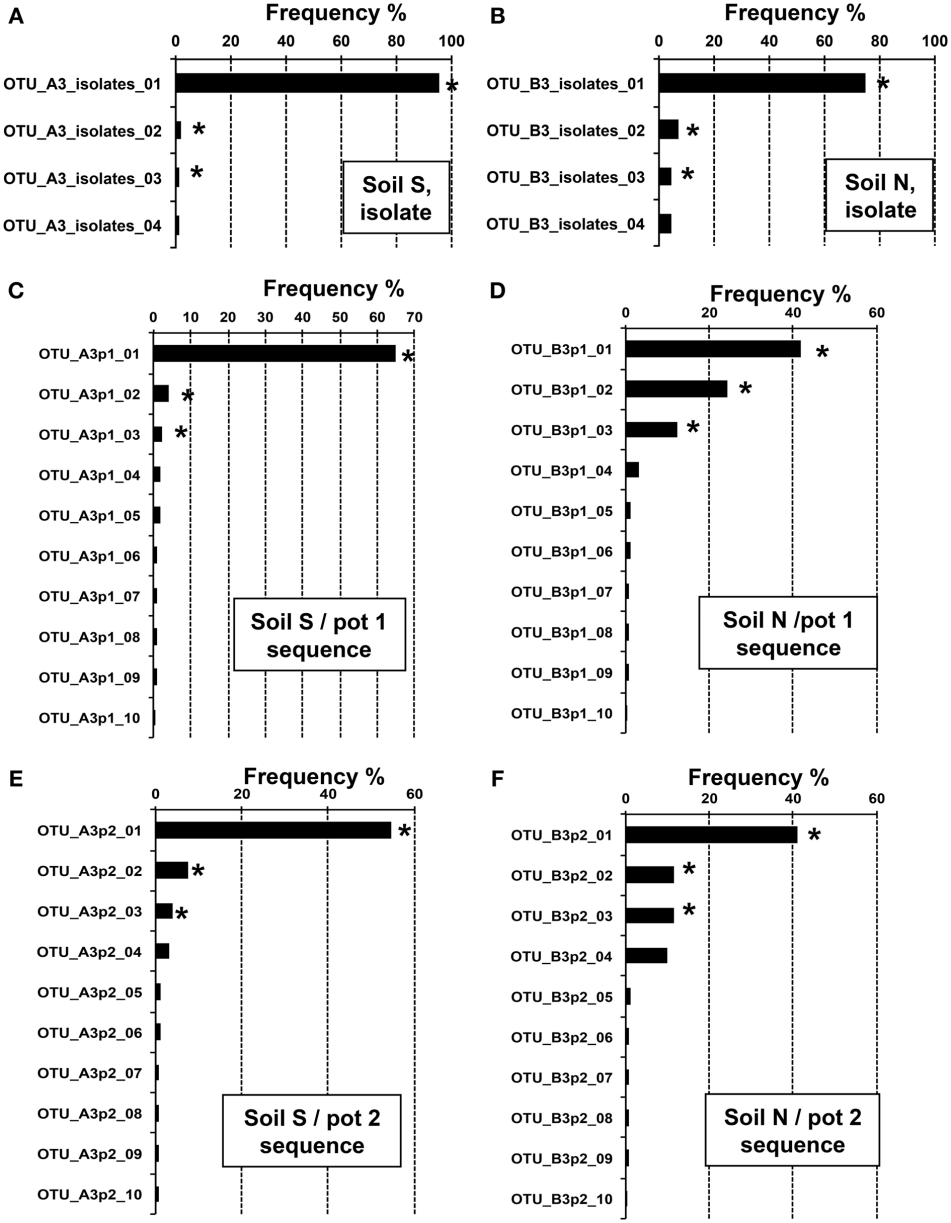
**Relative abundance of *Burkholderia* OTUs in MEP-sprayed soils.** Relative abundance of 16S rRNA gene sequences assigned to different OTUs (100% sequence identity thresholds) of the genus *Burkholderia* in the MEP-degrading isolates **(A,B)** and the deep sequence libraries **(C–F)**. **(A,C,E)** Soil S; **(B,D,F)** soil N. Data showed the top 4 or 10 OTUs with high frequency in *Burkholderia* isolates or sequences, respectively. OTUs with asterisks were subjected to phylogenetic analysis (see Figure 6).

To clarify the diversity of *Burkholderia* strains in the MEP-sprayed soils, 16S rRNA gene sequences of *Burkholderia* derived from the MEP-degrading isolates and the deep sequencing libraries of the soils treated with MEP three times (i.e., S3 and N3 soils) were classified into OTUs defined by 100% sequence identity. In the MEP-degrading isolates, 4 and 8 OTUs were identified from the S3 and N3 soils, respectively, in which a single and distinct OTU dominantly occupied at >70–90% frequency (Figures 5A,B). Although >100 OTUs were determined from all of the S3p1, S3p2, N3p1, and N3p2 libraries, a single and distinct OTU was dominant with >40% frequency in each soil sample (Figures 5C–F). In sequence libraries of both soil S and soil N, the dominant OTUs were not detected or a few sequences were detected before the MEP-treatment (Figure S3). These culture-dependent and -independent studies strikingly demonstrated that a particular phylotype of *Burkholderia* dominantly responds in soil environments in the presence of MEP.

**Figure 6:**
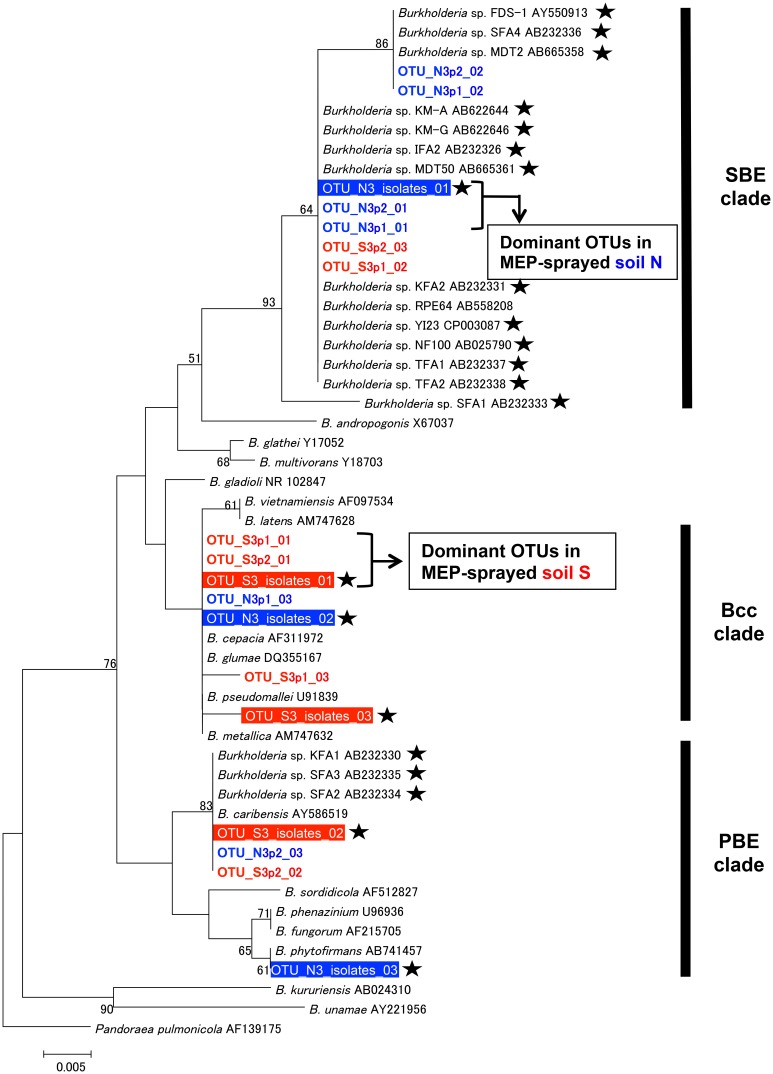
**Phylogenetic analysis of *Burkholderia* strains dominating in MEP-sprayed soils.** A neighbor-joining tree inferred from aligned 255 bp sequences of 16S rRNA gene is shown. Phylogenetic placements of OTUs (OTUs at 100% identity thresholds) of *Burkholderia*, identified from the MEP-degrading isolates and deep sequences, were estimated. Among dominant OTUs in each soil samples, top 3 OTUs (see Figure 5) were included in the analysis. Red and blue colored OTUs are detected from soil S and soil N, respectively. Outline letters on a filled background indicate OTUs determined by the isolation. Colored letters on a white background indicate OTUs detected by the deep sequencing. Asterisks indicate MEP-degrading *Burkholderia* strains. Clade names of SBE (stinkbug-associated beneficial and environmental group), Bcc (*Burkholderia cepacia* complex), and PBE (plant-associated beneficial and environmental group), respectively, according to Itoh et al. (2014), Coenye et al. (2001), and Suárez-Moreno et al. (2012), are shown on the right side. Bootstrap values higher than 50% are depicted at the nodes. The 16S rRNA sequence of *Pandoraea pulmonicola* (AF139175) was used as an outgroup.

We then estimated the phylogenetic relationship of these dominant *Burkholderia* strains based on 255 bp sequences of the V4 region of 16S rRNA gene (Figure 6). Regardless of whether the strain was identified by the culture-dependent or -independent method, each of the dominant *Burkholderia* OTUs derived from isolates and deep sequences was clustered together with the OTUs detected from the soil of the same origin (Figure 6), strongly indicating that our isolates are representative strains of MEP-degrading *Burkholderia* dominating the MEP-sprayed soils. This also indicates that the *Burkholderia* frequently detected from deep sequencing in MEP-sprayed soils are most likely to have MEP-degrading activity. The most dominant OTUs in soil S samples were related to pathogenic *Burkholderia* such as *B. cepacia*, *B. glumae*, and *B. pseudomallei* of the *Burkholderia cepacia* complex (Bcc) group (Coenye et al., 2001), while those in soil N samples were related to known MEP-degrading bacteria isolated from soils and symbiotic *Burkholderia* in stinkbugs (Tago et al., 2006; Kikuchi et al., 2011). These results suggest that the phylogenic lineage of *Burkholderia* responding to MEP-spraying appears likely to not be general, but specific for each soil environment.

## Discussion

### Effects of field-use history on soil microbiota responding to MEP treatment

Deep sequencing combined with culture-dependent approach clearly demonstrated that soil microbiota is strongly affected by the application of MEP, and furthermore, bacterial communities responding to MEP-spraying were remarkably different between the two andisol soils (soil S and soil N) (Figures 3, 4, 6 and Figure S2) despite their almost similar origin and chemical properties (Table S1). Notably, the two soils have different histories of insecticide application: the agricultural field where soil S was obtained had experienced at least 4 years of MEP spraying; the field where soil N was collected was not subjected to such insecticide application (Table S1). Therefore, in soil S, MEP-degrading bacteria (i.e., *Burkholderia* species) might already exist at a certain level, thus allowing them to respond quickly to the presence of the chemical. Meanwhile, the greater variety of MEP-degrading bacteria in soil N may be due to the absence of selection pressure from MEP. Although the enhanced biodegradation caused by repeated treatment with the same insecticide were reported from the 1970s (Felsot, 1989), little is known about the relation between soil microbial succession/adaptation and insecticide-spraying history. This is the first report to show the different microbial-community responses against insecticide re-treatment in soils that previously experienced such chemical application vs. naive soil with no such treatment prior to the experiment.

It should be noted that the bacterial composition of the initial states in both soils S and N was similar to each other (Figure S2) despite their different MEP-spraying history. This result strongly suggests that, although speculative, microbial community-structure disturbed by insecticide-spraying could recover to an initial community composition once the insecticide application is terminated. To clarify the plasticity of microbial community in more detail, it would be of great interest to investigate succession process of insecticide-enriched microbiota after terminating insecticide spraying. Interestingly, our results demonstrated that soil diagnostics of biological activity should not be inferred only based on a snapshot of environmental microbial community. To estimate potentials of a soil (microbiota) more precisely, it may be more important to trace the microbial succession under environmental stimuli.

### Burkholderia is a key player in MEP-degradation

Among the bacterial groups that dominantly responded to this insecticide, the genus *Burkholderia* was the most abundant after MEP-treatment (Figures 3C,D) and included a number of MEP-degrading isolates (Figure 2). Previous studies from China, Japan, and South Korea that were based on culture-dependent methods have repeatedly isolated MEP-degrading *Burkholderia* strains from MEP-contaminated soils at a higher frequency relative to other genera (Tago et al., 2006; Zhang et al., 2006; Kim et al., 2009). Taken together, our findings from both culture-dependent and -independent approaches strongly suggest that *Burkholderia* is a key bacterial group for MEP-degradation in soil environments. Remarkably, *Burkholderia* species were also found to degrade other organophosphorus insecticides, which includes parathion, methyl parathion, glyphosate, and chlorpyrifos-methyl (Keprasertsupa et al., 2001; Kuklinsky-Sobral et al., 2005; Kim et al., 2007b, 2009). Considering that MEP-degrading strains of *Burkholderia* frequently show cross-acclimation against other organophosphorus compounds (Hayatsu et al., 2000; Kim and Ahn, 2009; Kim et al., 2009; Kikuchi et al., 2012), *Burkholderia* might have a pivotal role in the degradation of various insecticides. To clarify this point, it would be of great interest to also employ a deep sequencing survey of 16S rRNA genes for various types of organophosphorus insecticide-contaminated soils.

### Other bacterial groups responding to MEP-spraying

Previous studies have isolated and characterized MEP-degrading bacteria from genera *Cupriavidus*, *Pseudomonas*, *Sphingomonas*, *Corynebacterium*, *Arthrobacter*, and *Burkholderia* (Tago et al., 2006; Zhang et al., 2006; Kim et al., 2009). In addition to *Burkholderia*, members of genera *Dyella*, *Ralstonia*, *Pandoraea*, and *Achromobacter* were also isolated as MEP-degrading bacteria in our culture-dependent experiments (Figure 2). Hence, this is the first record of MEP-degrading strains from these four genera, suggesting that broader taxonomic groups could contribute to MEP-degradation in natural soil environments. Some strains of *Ralstonia* and *Achromobacter* have already been reported to degrade organophosphorus compounds that are analogous to MEP, particularly fenamifos (organophosphorus nematicide) and methyl parathion (organophosphorus insecticide), respectively, (Zhang et al., 2005; Cabrera et al., 2010). Similar to *Burkholderia* strains, these bacteria probably have an ability for cross-acclimation toward other organophosphorus compounds.

In addition to the complete degradation of MEP by a single bacterium, the cooperative degradation of MEP has also been reported *in vitro*. In this case, MEP-hydrolyzing and MEP-hydrolysate-degrading bacteria cooperate for complete consumption of the chemical compound (Katsuyama et al., 2009). Previous studies have shown that some *Burkholderia* strains, though ineffective against MEP, are able to degrade its hydrolysate, 3-methyl-4-nitrophenol (Kim et al., 2007a; Katsuyama et al., 2009). Hence, some of the increasing bacteria identified by deep sequencing (Figure 4) from the MEP-sprayed soils might be directly involved in the hydrolysis of MEP, while others might contribute to the degradation of the resulting hydrolysates. Such a synergistic interaction among bacterial species could promote efficient degradation of MEP in soils.

### Methylotrophs could play a role in MEP-degradation

**Figure 7:**
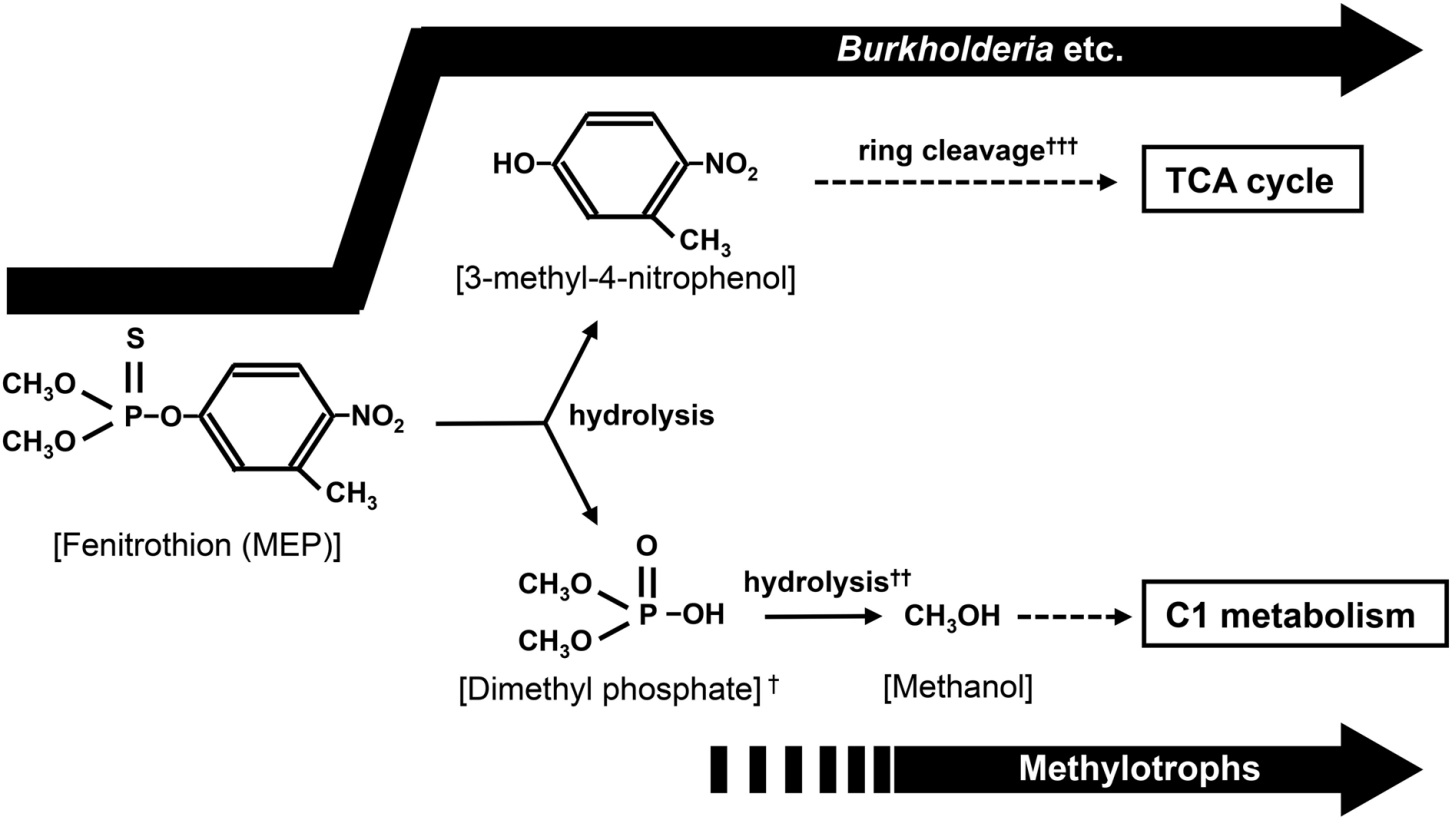
**Proposed biodegradation pathway of MEP.** In addition to the utilization of 3-methyl-4-nitrophenole by *Burkholderia* and other bacteria, methanol generated from hydrolysis of dimethyl phosphate might be used as a carbon source by soil bacteria like methylotrophs. ^†^P=S was rapidly substituted to P=O by oxidation in environment (Bajgar, 2004; Karpouzas and Singh, 2006). ^††^(CH_3_O)_2_POOH + 2H_2_O → 2CH_3_OH + PO^3−^_4_ + 3H^+^. ^†††^ The ring cleavage process has been reported in the biodegradation of MEP or MEP analog (Hayatsu et al., 2000; Zhang et al., 2006).

Notably, deep sequencing revealed that the frequency of *Methylobacillus*, *Methylobacterium*, and *Methylophilus*, which were never detected by the culture-dependent approach in this study and from previous researches (Tago et al., 2006; Zhang et al., 2006; Kim et al., 2009), also dramatically increased in soils after MEP-spraying (Figure 4). Most members of these genera are methylotrophic, that is, they actively use one-carbon compounds such as methanol as a sole carbon source (Kolb and Stacheter, 2013). Past studies have demonstrated that bacteria break down MEP into 3-methyl-4-nitrophenol and dimethyl phosphate during the initial hydrolysis step (Spillner et al., 1979; IPCS, 1992) (Figure 7). Though the succeeding stages for biodegrading the former aromatic compound have been described in detail (Spillner et al., 1979; IPCS, 1992; Hayatsu et al., 2000), the fate of dimethyl phosphate in soil still remains unclear. In the case of the degradation of paraoxon, an analog of MEP, the initial hydrolysis process produces diethyl phosphate, which is then further hydrolyzed into two molecules of ethanol by *Delftia acidovorans* and *Pseudomonas putida* through phosphodiesterase and phosphatase enzymes (Tehara and Keasling, 2003). Although speculative, dimethyl phosphate may also be hydrolyzed into two molecules of methanol by a similar route during MEP-degradation. If so, the methylotrophs detected in this study may consume and utilize such generated methanol for growth (Figure 7).

It has been reported that accumulated methanol is harmful to organisms that are unable to metabolize it (Martin-Amat et al., 1978). In fact, growth of MEP-degrading *Burkholderia* strains isolated in this study was depressed by a tiny amount of methanol contamination (Figure S4). If methylotrophs rapidly consume any methanol generated during MEP-degradation, they might protect other bacteria involved in the overall degradation process from the toxicity of this by-product. This could probably lead to better growth of such relevant bacterial communities, thus contributing to more efficient and complete mineralization of MEP. This hypothesis could be verified through future evaluations on the (1) degradability of dimethyl phosphate by methylotrophs and (2) co-culture of MEP-degrading bacteria and methylotrophs.

## Concluding remarks

This study demonstrated the effect of MEP-spraying history to the microbial succession under MEP application and identified key bacteria for MEP-degradation by performing both culture experiments and high-throughput sequencing. Owing to the enormous information generated by the deep sequencing of bacterial 16S rRNA gene, we comprehensively revealed the community structures of bacterial species responding to MEP, and found previously unseen members, methylotrophs, that may play an important role in the complete degradation of MEP in soil environments. In contrast, it should be noted that the partial sequencing of 16S rRNA gene gave us only indirect information about the MEP-degrading strains. In fact, our previous study reported that even *Burkholderia* strains exhibiting high (>99.8%) sequence similarities in 16S rRNA gene possess quite different MEP-degrading activities (Kikuchi et al., 2012). Sequencing of functional genes involved in the MEP-degradation pathway might give more direct information for MEP-degradation in environmental soils, although MEP-degradation genes are not well understood (Singh, 2009). In addition, fungal biodegradation of MEP has been also reported (Baarschers and Heitland, 1986), although here we focused only on bacteria. Metagenomic approaches of functional genes and more comprehensive survey including fungal communities in soils should improve our knowledge of MEP-degradation by soil microorganisms.

### Conflict of interest statement

The authors declare that the research was conducted in the absence of any commercial or financial relationships that could be construed as a potential conflict of interest.

## References

[B1] Arbeli Z. Fuentes L. C. (2007). Accelerated biodegradation of pesticides: an overview of the phenomenon, its basis and possible solutions; and a discussion on the tropical dimension. Crop Protect. 26, 1733–1746 10.1016/j.cropro.2007.03.009

[B2] Arora P. K. Srivastava A. Singh V. P. (2013). Bacterial degradation of nitrophenols and their derivatives. J. Hazard. Mater. 266C, 42–59 10.1016/j.jhazmat.2013.12.011 24374564

[B3] Baarschers H. W. Heitland S. H. (1986). Biodegradation of fenitrothion and fenitrooxon by the fungus *Trichoderma viride*. J. Agric. Food Chem. 34, 707–709 10.1021/jf00070a029

[B4] Bajgar J. (2004). Organophosphates/nerve agent poisoning: mechanism of action, diagnosis, prophylaxis, and treatment. Adv. Clin. Chem. 38, 151–216 10.1016/S0065-2423(04)38006-6 15521192

[B5] Cabrera J. A. Kurtz A. Sikora R. A. Schouten A. (2010). Isolation and characterization of fenamiphos degrading bacteria. Biodegradation 21, 1017–1027 10.1007/s10532-010-9362-z 20464454

[B6] Caporaso J. G. Kuczynski J. Stombaugh J. Bittinger K. Bushman F. D. Costello E. K. (2010). QIIME allows analysis of high-throughput community sequencing data. Nat. Methods 7, 335–336 10.1038/nmeth.f.303 20383131 PMC3156573

[B7] Caporaso J. G. Lauber C. L. Walters W. A. Berg-Lyons D. Huntley J. Fierer N. (2012). Ultra-high-throughput microbial community analysis on the Illumina HiSeq and MiSeq platforms. ISME J. 6, 1621–1624 10.1038/ismej.2012.8 22402401 PMC3400413

[B8] Coenye T. Vandamme P. Govan J. R. LiPuma J. J. (2001). Taxonomy and identification of the *Burkholderia cepacia* complex. J. Clin. Microbiol. 39, 3427–3436 10.1128/JCM.39.10.3427-3436.2001 11574551 PMC88367

[B9] da Silva N. A. Birolli W. G. Seleghim M. H. R. Porto A. L. M. (2013). Biodegradation of the organophosphate pesticide profenofos by Marine Fungi, in Applied Bioremediation-Active and Passive Approaches, eds Patil Y. B. Rao P. (Rijeka: InTechWeb), 149–180 10.5772/56372

[B11] DeSantis T. Z. Hugenholtz P. Larsen N. Rojas M. Brodie E. L. (2006). Greengenes, a chimera-checked 16S rRNA gene database and workbench compatible with ARB. Appl. Environ. Microbiol. 72, 5069–5072 10.1128/AEM.03006-05 16820507 PMC1489311

[B12] Diez M. C. (2010). Biological aspects involved in the degradation of organic pollutants. J. Soil. Sci. Plant Nutr. 10, 244–267 10.4067/S0718-95162010000100004

[B13] Fang H. Cai L. Yang Y. Ju F. Li X. Yu Y. (2014). Metagenomic analysis reveals potential biodegradation pathways of persistent pesticides in freshwater and marine sediments. Sci. Total Environ. 1, 983–992 10.1016/j.scitotenv.2013.10.076 24239819

[B14] Felsot A. S. (1989). Enhanced biodegradation of insecticides in soil: implications for agroecosystems. Ann. Rev. Entomol. 34, 453–476 10.1146/annurev.en.34.010189.002321

[B16] Fukatsu T. Nikoh N. (1998). Two intracellular symbiotic bacteria from the mulberry psyllid *Anomoneura mori* (Insecta, Homoptera). Appl. Environ. Microbiol. 64, 3599–3606 9758773 10.1128/aem.64.10.3599-3606.1998 PMC106470

[B17] Hayatsu M. Hirano M. Tokuda S. (2000). Involvement of two plasmids in MEP degradation by *Burkholderia* sp.strain NF100. Appl. Environ. Microbiol. 66, 1737–1740 10.1128/AEM.66.4.1737-1740.2000 10742273 PMC92054

[B19] International Programme on Chemical Safety (IPCS). (1992). Environmental health criteria 133: fenitrothion, in World Health Organization (Accessed December 22, 2013). http://www.inchem.org/documents/ehc/ehc/ehc133.htm

[B20] Itoh H. Aita M. Nagayama A. Meng X. Y. Kamagata Y. Navarro R. (2014). Evidence of environmental and vertical transmission of *Burkholderia* symbionts in the oriental chinch bug *Cavelerius saccharivorus* (Heteroptera: Blissidae). Appl. Environ. Microbiol. (in press). 10.1128/AEM.01087-14 25038101 PMC4178689

[B21] Jacobsen C. S. Hjelmsø M. H. (2014). Agricultural soils, pesticides and microbial diversity. Curr. Opin. Biotech. 27, 15–20 10.1016/j.copbio.2013.09.003 24863892

[B22] Karpouzas D. G. Singh B. K. (2006). Microbial degradation of organophosphorus xenobiotics: metabolic pathways and molecular basis. Adv. Microb. Physiol. 51, 119–185 10.1016/S0065-2911(06)51003-3 17091564

[B23] Katsuyama C. Nakaoka S. Takeuchi Y. Tago K. Hayatsu M. Kato K. (2009). Complementary cooperation between two syntrophic bacteria in pesticide degradation. J. Theor. Biol. 256, 644–654 10.1016/j.jtbi.2008.10.024 19038271

[B24] Keprasertsupa C. Upathamb E. S. Sukhapanthb N. Prempreed P. (2001). Degradation of methyl parathion in an aqueous medium by soil Bacteria. Scienceasia 27, 261–270 10.2306/scienceasia1513-1874.2001.27.261

[B25] Kikuchi Y. Hayatsu M. Hosokawa T. Nagayama A. Tago K. Fukatsu T. (2012). Symbiont-mediated insecticide resistance. Proc. Natl. Acad. Sci. U.S.A. 109, 8618–8622 10.1073/pnas.1200231109 22529384 PMC3365206

[B26] Kikuchi Y. Hosokawa T. Fukatsu T. (2011). An ancient but promiscuous host-symbiont association between *Burkholderia* gut symbionts and their heteropteran hosts. ISME J. 5, 446–460 10.1038/ismej.2010.150 20882057 PMC3105724

[B27] Kim J. R. Ahn Y. J. (2009). Identification and characterization of chlorpyrifos-methyl and 3,5,6-trichloro-2-pyridinol degrading *Burkholderia* sp. strain KR100. Biodegradation 20, 487–497 10.1007/s10532-008-9238-7 19082866

[B28] Kim K. D. Ahn J. H. Kim T. Park S. C. Seong C. N. Song H. G. (2009). Genetic and phenotypic diversity of fenitrothion-degrading bacteria isolated from soils. J. Microbiol. Biotechnol. 19, 113–120 10.4014/jmb.0808.467 19307758

[B29] Kim S. H. Park M. R. Han S. Whang K. Shim J. H. Kim I. S. (2007a). Degradation of 3-Methyl-4-nitrophenol, a Main Product of the Insecticide Fenitrothion, by *Burkholderia* sp. SH-1 Isolated from Earthworm (*Eisenia fetida*) Intestine. J. Appl. Biol. Chem. 50, 281–287

[B30] Kim T. Ahn J. H. Choi M. K. Weon H. Y. Kim M. S. Seong C. N. (2007b). Cloning and expression of a parathion hydrolase gene from a soil bacterium, *Burkholderia* sp. JBA3. J. Microbiol. Biotechnol. 17, 1890–1893 18092477

[B31] Kolb S. Stacheter A. (2013). Prerequisites for amplicon pyrosequencing of microbial methanol utilizers in the environment. Front. Microbiol. 4:268 10.3389/fmicb.2013.00268 24046766 PMC3763247

[B32] Kuklinsky-Sobral J. Araújo W. L. Mendes R. Pizzirani-Kleiner A. Azevedo J. (2005). Isolation and characterization of endophytic bacteria from soybean (*Glycine max*) grown in soil treated with glyphosate herbicide. Plant Soil 273, 91–99 10.1007/s11104-004-6894-1

[B33] Martin-Amat G. McMartin K. E. Hayreh S. S. Hayreh M. S. Tephly T. R. (1978). Methanol poisoning: ocular toxicity produced by formate. Toxicol. Appl. Pharmacol. 45, 201–208 10.1016/0041-008X(78)90040-6 99844

[B34] Meyer F. Paarmann D. D'Souza M. Olson R. Glass E. M. Kubal M. (2008). The metagenomics RAST server - a public resource for the automatic phylogenetic and functional analysis of metagenomes. BMC Bioinformatics 9:386 10.1186/1471-2105-9-386 18803844 PMC2563014

[B35] Moorthie S. Mattocks C. J. Wright C. F. (2011). Review of massively parallel DNA sequencing technologies. Hugo J. 5, 1–12 10.1007/s11568-011-9156-3 23205160 PMC3238019

[B56] Muyzer G. de Waal E. C. Uitterlinden A. G. (1993). Profiling of complex microbial populations by denaturing gradient gel electrophoresis analysis of polymerase chain reaction-amplified genes coding for 16S rRNA. Appl. Environ. Microbiol. 59, 695–700 7683183 10.1128/aem.59.3.695-700.1993 PMC202176

[B36] Noll M. Matthies D. Frenzel P. Derakshani M. Liesack W. (2005). Succession of bacterial community structure and diversity in a paddy soil oxygen gradient. Environ. Microbiol. 7, 382–395 10.1111/j.1462-2920.2005.00700.x 15683399

[B37] O'Connell J. L. Nyman J. A. (2011). Effects of marsh pond terracing on coastal wintering waterbirds before and after Hurricane Rita. Environ. Manage. 48, 975–984 10.1007/s00267-011-9741-1 21874599

[B38] Peñuelas J. Sardans J. Estiarte M. Ogaya R. Carnicer J. Coll M. (2013). Evidence of current impact of climate change on life: a walk from genes to the biosphere. Glob. Chang. Biol. 19, 2303–2338 10.1111/gcb.12143 23505157

[B39] Phillips O. L. Aragão L. E. Lewis S. L. Fisher J. B. Lloyd J. López-González G. (2009). Drought sensitivity of the Amazon rainforest. Science 323, 1344–1347 10.1126/science.1164033 19265020

[B40] R Development Core Team. (2008). R: A Language and Environment for Statistical Computing. R Foundation for Statistical Computing. http://www.R-project.org

[B41] Rota C. T. Millspaugh J. J. Rumble M. A. Lehman C. P. Kesler D. C. (2014). The role of wildfire, prescribed fire, and mountain pine beetle infestations on the population dynamics of black-backed woodpeckers in the black hills, South dakota. PLoS ONE 9:e94700 10.1371/journal.pone.0094700 24736502 PMC3988106

[B42] Schloss P. D. Westcott S. L. Ryabin T. Hall J. R. Hartmann M. Hollister E. B. (2009). Introducing mothur: open-source, platform-independent, community-supported software for describing and comparing microbial communities. Appl. Environ. Microbiol. 75, 7537–7541 10.1128/AEM.01541-09 19801464 PMC2786419

[B44] Singh B. K. (2009). Organophosphorus-degrading bacteria: ecology and industrial applications. Nat. Rev. Microbiol. 7, 156–164 10.1038/nrmicro2050 19098922

[B45] Singh B. K. Walker A. (2006). Microbial degradation of organophosphorus compounds. FEMS Microbiol. Rev. 30, 428–471 10.1111/j.1574-6976.2006.00018.x 16594965

[B46] Spillner C. J. DeBaun J. R. Menn J. J. (1979). Degradation of fenitrothion in forest soil and effects on forest soil microbes. J. Agric. Food Chem. 27, 1054–1060 10.1021/jf60225a009

[B47] Suárez-Moreno Z. R. Caballero-Mellado J. Coutinho B. G. Mendonça-Previato L. James E. K. (2012). Common features of environmental and potentially beneficial plant-associated *Burkholderia*. Microb. Ecol. 63, 249–266 10.1007/s00248-011-9929-1 21850446

[B48] Tago K. Sekiya E. Kiho A. Katsuyama C. Hoshito Y. Yamada N. (2006). Diversity of fenitrothion-degrading bacteria in soils from distant geographical areas. Microbes Environ. 21, 58–64 10.1264/jsme2.21.58

[B49] Tamura K. Dudley J. Nei M. Kumar S. (2007). MEGA4: molecular Evolutionary Genetics Analysis (MEGA) software version 4.0. Mol. Biol. Evol. 24, 1596–1599 10.1093/molbev/msm092 17488738

[B50] Tehara S. K. Keasling J. D. (2003). Gene cloning, purification, and characterization of a phosphodiesterase from *Delftia acidovorans*. Appl. Environ. Microbiol. 9, 504–508 10.1128/AEM.69.1.504-508.2003 12514034 PMC152426

[B51] Wang Q. Garrity G. M. Tiedje J. M. Cole J. R. (2007). Naïve bayesian classifier for rapid assignment of rRNA Sequences into the new bacterial taxonomy. Appl. Environ. Microbiol. 73, 5261–5267 10.1128/AEM.00062-07 17586664 PMC1950982

[B52] Whalon M. E. Monte-Sanchez D. Hollingworth R. M. (2008). Global Pesticide Resistance in Arthropods. London: CAB International 10.1079/9781845933531.0000

[B53] Wilson M. C. Piel J. (2013). Metagenomic approaches for exploiting uncultivated bacteria as a resource for novel biosynthetic enzymology. Chem. Biol. 20, 636–647 10.1016/j.chembiol.2013.04.011 23706630

[B54] Zhang R. Cui Z. Jiang J. He J. Gu X. Li S. (2005). Diversity of organophosphorus pesticide-degrading bacteria in a polluted soil and conservation of their organophosphorus hydrolase genes. Can. J. Microbiol. 51, 337–343 10.1139/w05-010 15980896

[B55] Zhang Z. Hong Q. Xu J. Zhang X. Li S. (2006). Isolation of fenitrothion-degrading strain *Burkholderia* sp. FDS-1 and cloning of mpd gene. Biodegradation 17, 275–283 10.1007/s10532-005-7130-2 16715406

