# Supplementary material for: Bacterial population succession and adaptation affected by insecticide application and soil spraying history
Source: Front Microbiol. 2014 Aug 29;5:457. doi: 10.3389/fmicb.2014.00457 (PMC4148734; doi:10.3389/fmicb.2014.00457)
Supplement: Supplementary file 1 [file DataSheet1.PDF]

Supplementary Material

Bacterial population succession and adaptation affected by insecticide application and soil spraying history

Hideomi Itoh[1], Ronald Navarro[2], Kazutaka Takeshita[1], Kanako Tago[3], Masahito Hayatsu[3], Tomoyuki Hori[2], Yoshitomo Kikuchi[1]*

[1] Bioproduction Research Institute, National Institute of Advanced Industrial Science and Technology (AIST), Sapporo, Japan
[2] Research Institute for Environmental Management Technology, National Institute of Advanced Industrial Science and Technology (AIST), Tsukuba, Japan
[3] Environmental Biofunction Division, National Institute for Agro-Environmental Sciences (NIAES), Tsukuba, Japan

*** Correspondence:** Dr. Yoshitomo Kikuchi, National Institute of Advanced Industrial Science and Technology (AIST), Bioproduction Research Institute, 2-17-2-1, Tsukisamu-higashi, Toyohira-ku, Sapporo, Hokkaido 062-8517, Japan
y-kikuchi@aist.go.jp

Supplementary Figures and Tables

Figrue S1. A schematic of sampling points of insecticide-sprayed soils in this study. Soil samples were collected from each MEP-treated pot loaded with soil S (S0, S1, S2, S3) or N (N0, N1, N2, N3) every week and subjected to (a) CFU counting, (b) isolation of MEP-degrading bacteria, (c) deep sequencing, and (d) qPCR. As an experimental control, distilled water was sprayed once a week and soils were collected one week after 3rd spraying. (S3C and N3C). The spraying experiments were duplicated (pot 1 and pot 2) and all of obtained samples were subjected to the above experiments.

Figure S2. Principal coordinate analysis (PCoA) of the microbiotas in MEP-sprayed soils. PCoA plots were generated by the weighted unifrac analysis based on the same amount of sequences (6,218 sequences) of each library. Description of library IDs is listed in Table 1. Black and gray plots represent libraries derived from soil S and soil N, respectively. The values in parentheses show the percentage of community variation explained by each coordinate. Arrows indicate the temporal transition sequences.

Figure S3. Phylogenetic analysis of *Burkholderia* sequences derived from soils before and after MEP treatment. Enclosed IDs and number in parenthesis indicate the sequences derived from soils before MEP treatment and number of sequences, respectively. Details were showed in legend of Figure 6.

Figure S4. Effects of methanol on growth of MEP-degrading *Burkholderia* strains. (A) OTU_S3_isolates_01 and (B) OTU_N3_isolates_01, isolated from soil S3 and N3, respectively. These strains were cultured in BSYG medium (0.1% glucose, 20 mM potassium phosphate (pH 7.0), 0.1% $(NH_4)_2SO_4$, 0.02% NaCl, 0.01% $MgSO_4$•$7H_2O$, 0.05% $CaCl_2$•$2H_2O$, 0.0002% $FeSO_4$•$7H_2O$, 0.1% yeast extract) containing 5% (squares) or 1% (triangles) methanol, or none (circles). Note the OTU_S3_isolates_01 and OTU_N3_isolates_01 dominated in MEP-sprayed soils (Figure 5&6).

Table S1 Chemical properties of soil samples used in this study.

Table S2 Identification of MEP-degrading bacteria isolated in this study.

Figure S1

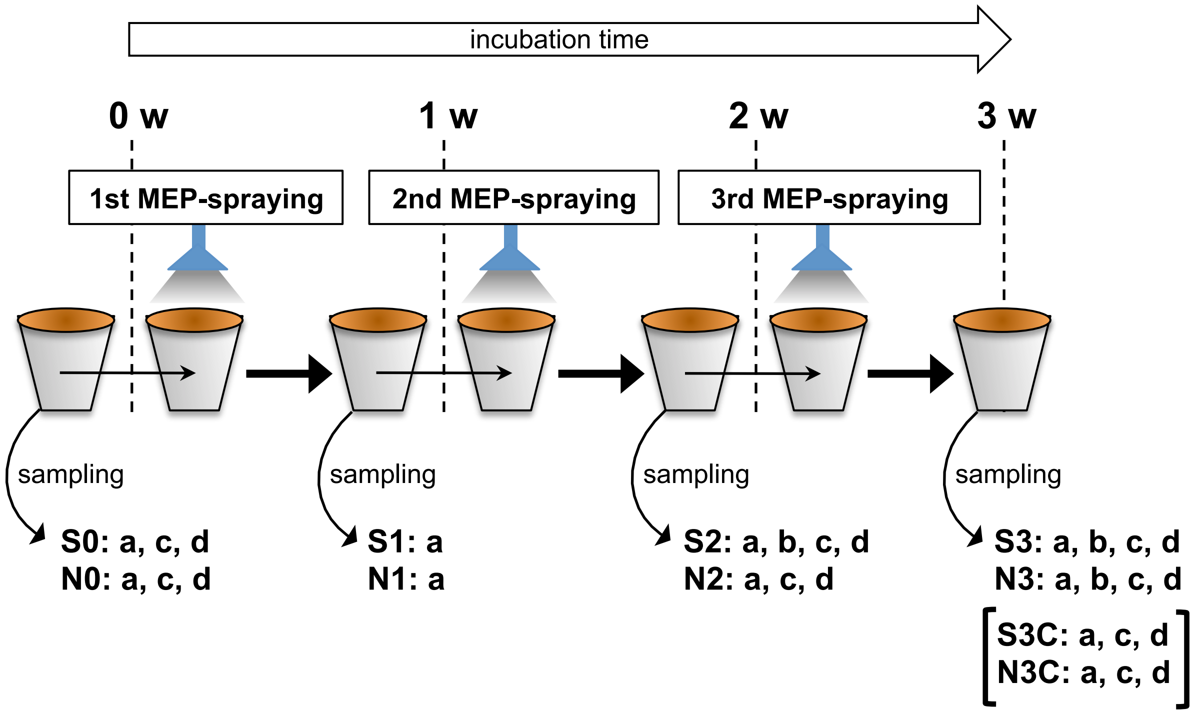

incubation time
0 w
1 w
2 w
3 w
1st MEP-spraying
2nd MEP-spraying
3rd MEP-spraying
sampling
sampling
sampling
sampling
S0: a, c, d
N0: a, c, d
S1: a
N1: a
S2: a, b, c, d
N2: a, c, d
S3: a, b, c, d
N3: a, b, c, d
S3C: a, c, d
N3C: a, c, d

Figure S2

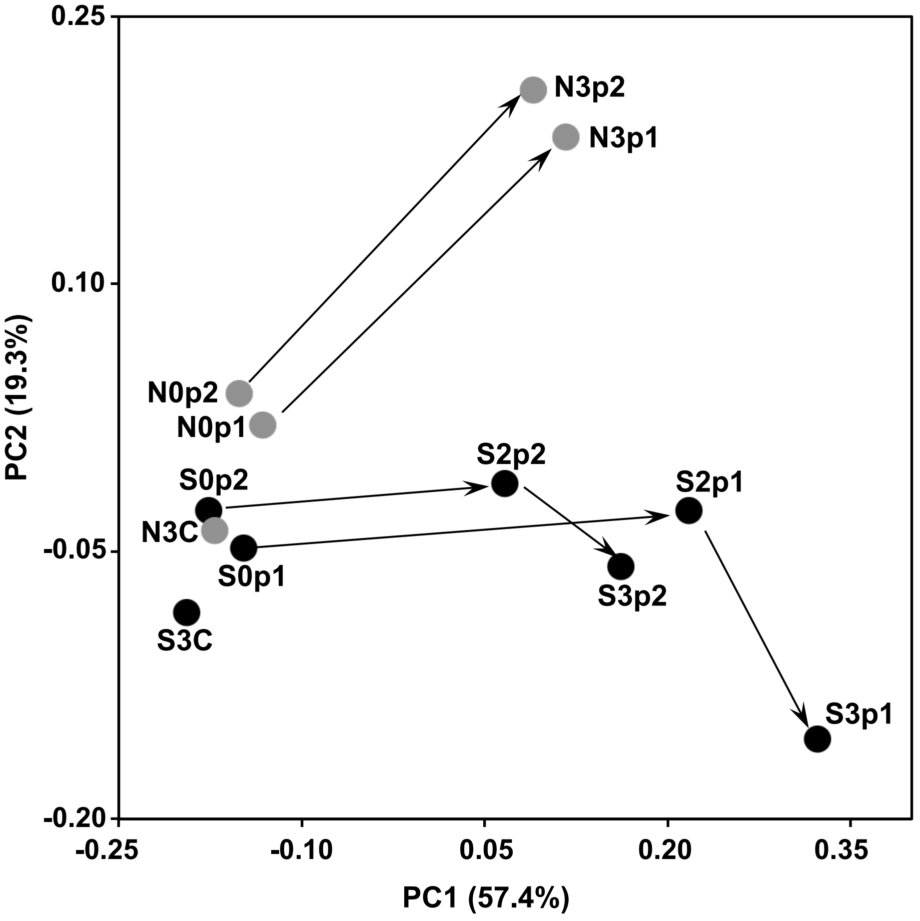

0.25
0.10
-0.05
-0.20
-0.25
-0.10
0.05
0.20
0.35
PC1 (57.4%)
PC2 (19.3%)
N3p2
N3p1
N0p2
N0p1
S2p2
S0p2
S2p1
N3C
S0p1
S3p2
S3C
S3p1

Figure S3

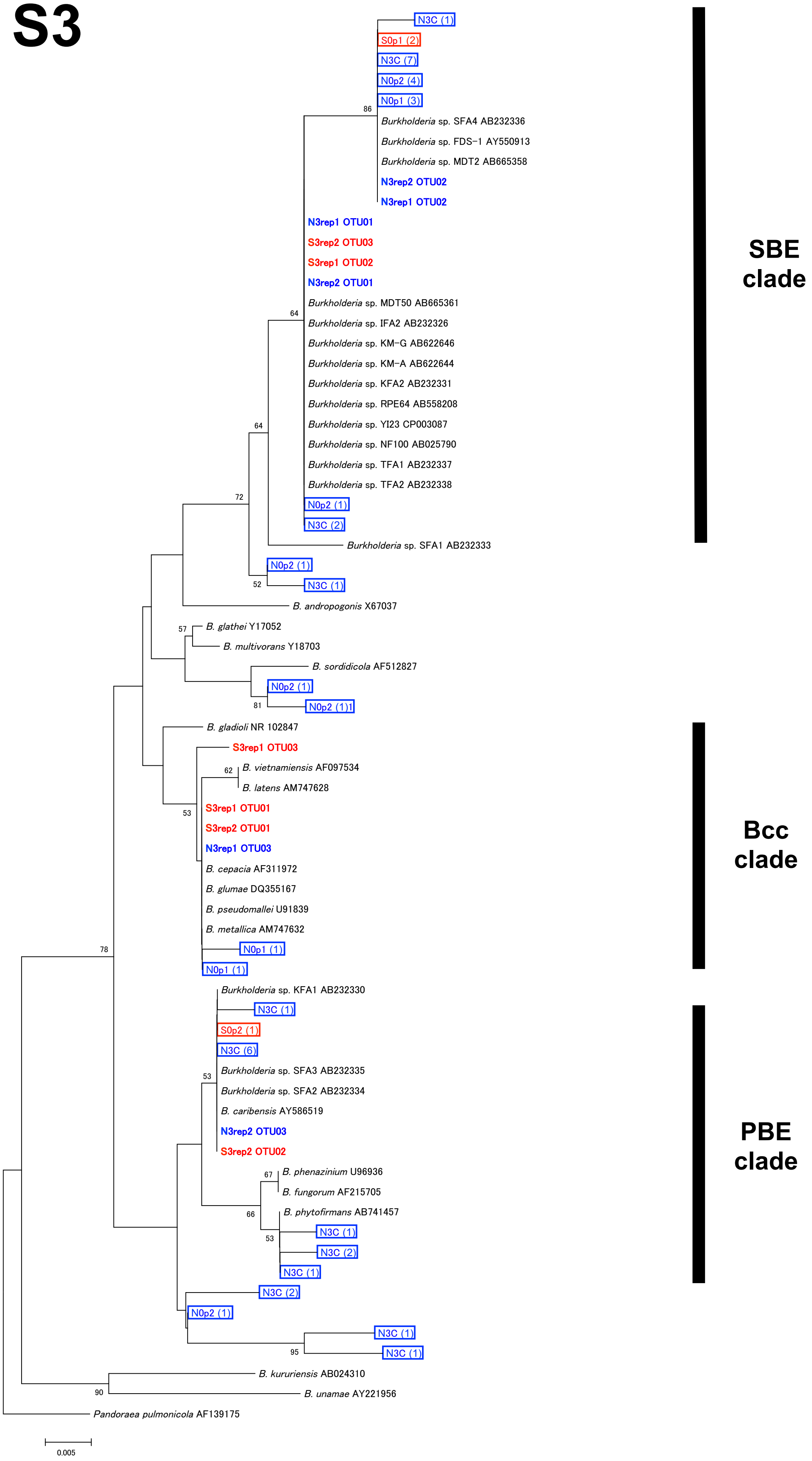

N3C (1)
S0p1 (2)
N3C (7)
N0p2 (4)
N0p1 (3)
86
Burkholderia sp. SFA4 AB232336
Burkholderia sp. FDS-1 AY550913
Burkholderia sp. MDT2 AB665358
N3rep2 OTU02
N3rep1 OTU02
N3rep1 OTU01
S3rep2 OTU03
S3rep1 OTU02
N3rep2 OTU01
Burkholderia sp. MDT50 AB665361
64
Burkholderia sp. IFA2 AB232326
Burkholderia sp. KM-G AB622646
Burkholderia sp. KM-A AB622644
Burkholderia sp. KFA2 AB232331
Burkholderia sp. RPE64 AB558208
64
Burkholderia sp. YI23 CP003087
Burkholderia sp. NF100 AB025790
Burkholderia sp. TFA1 AB232337
Burkholderia sp. TFA2 AB232338
72
N0p2 (1)
N3C (2)
Burkholderia sp. SFA1 AB232333
N0p2 (1)
52
N3C (1)
B. andropogonis X67037
57
B. glathei Y17052
B. multivorans Y18703
B. sordidicola AF512827
N0p2 (1)
81
N0p2 (1)1
B. gladioli NR 102847
S3rep1 OTU03
62
B. vietnamiensis AF097534
B. latens AM747628
53
S3rep1 OTU01
S3rep2 OTU01
N3rep1 OTU03
B. cepacia AF311972
B. glumae DQ355167
B. pseudomallei U91839
B. metallica AM747632
78
N0p1 (1)
N0p1 (1)
Burkholderia sp. KFA1 AB232330
N3C (1)
S0p2 (1)
N3C (6)
53
Burkholderia sp. SFA3 AB232335
Burkholderia sp. SFA2 AB232334
B. caribensis AY586519
N3rep2 OTU03
S3rep2 OTU02
67
B. phenazinium U96936
B. fungorum AF215705
66
B. phytofirmans AB741457
53
N3C (1)
N3C (2)
N3C (1)
N3C (2)
N0p2 (1)
N3C (1)
95
N3C (1)
B. kururiensis AB024310
90
B. unamae AY221956
Pandoraea pulmonicola AF139175
0.005
SBE clade
Bcc clade
PBE clade

Figure S4

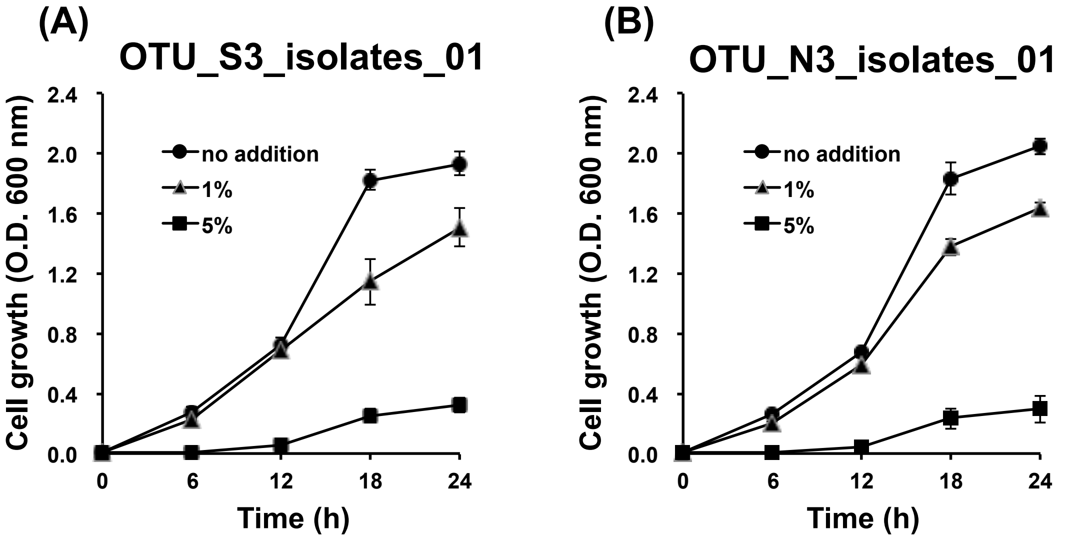
(A)
OTU_S3_isolates_01
Cell growth (O.D. 600 nm)
2.4
2.0
1.6
1.2
0.8
0.4
0.0
no addition
1%
5%
0
6
12
18
24
Time (h)
(B)
OTU_N3_isolates_01
Cell growth (O.D. 600 nm)
2.4
2.0
1.6
1.2
0.8
0.4
0.0
no addition
1%
5%
0
6
12
18
24
Time (h)

Table S1. Chemical properties and MEP-spraying history of soil samples investigated in this study.

Soil	Soil taxonomy	MEP history	Water content (w/w, %)	pH (H2O)	Total C (%)	Total N (%)
S	humic andisol	sprayed[a]	35.2	6.3	5.0	N.D.[c]
N	humic andisol	naive[b]	33.7	6.6	4.4	N.D.[c]

[a] Once or more a year from 2009 to 2012.

[b] MEP and any other chemical compounds were not sprayed for at least last five years.

[c] Not detected (<0.3%).

Table S2 Identification of MEP-degrading bacteria isolated in this study.

Orgin soil[a]	Strain ID [Accession No.]	Length (bp)	RDP classifier		BlastN		
			Closest genus	Confidence	Top hit sequences	Accession No.	Identity (%)
S2	IBa201 [AB904935]	623	*Burkholderia*	1	*Burkholderia cenocepacia*	GU433447	100
S2	IBa202 [AB904936]	618	*Burkholderia*	1	*Burkholderia cenocepacia*	FJ870663	100
S2	IBa203 [AB904937]	616	*Burkholderia*	1	*Burkholderia cenocepacia*	FJ870663	99.51
S2	IBa204 [AB904938]	623	*Burkholderia*	1	*Burkholderia cenocepacia*	GU433447	99.68
S2	IBa205 [AB904939]	614	*Burkholderia*	1	*Burkholderia cenocepacia*	FJ870663	100
S2	IBa206 [AB904940]	615	*Burkholderia*	1	*Burkholderia cenocepacia*	FJ870663	100
S2	IBa207 [AB904941]	629	*Burkholderia*	1	*Burkholderia cenocepacia*	GU433447	100
S2	IBa208 [AB904942]	620	*Burkholderia*	1	*Burkholderia cenocepacia*	GU433447	99.68
S2	IBa209 [AB904943]	621	*Burkholderia*	1	*Burkholderia cenocepacia*	GU433447	99.84
S2	IBa210 [AB904944]	625	*Burkholderia*	1	*Burkholderia cenocepacia*	GU433447	100
S2	IBa211 [AB904945]	622	*Burkholderia*	1	*Burkholderia cenocepacia*	GU433447	100
S2	IBa212 [AB904946]	615	*Burkholderia*	1	*Burkholderia cenocepacia*	FJ870663	99.84
S2	IBa213 [AB904947]	617	*Burkholderia*	1	*Burkholderia* sp.	FJ560464	100
S2	IBa214 [AB904948]	619	*Burkholderia*	1	*Burkholderia cenocepacia*	GU433447	100
S2	IBa215 [AB904949]	620	*Burkholderia*	1	*Burkholderia cenocepacia*	GU433447	99.84
S2	IBa216 [AB904950]	626	*Burkholderia*	1	*Burkholderia cenocepacia*	GU433447	100
S2	IBa217 [AB904951]	622	*Burkholderia*	1	*Burkholderia cenocepacia*	GU433447	100
S2	IBa218 [AB904952]	632	*Burkholderia*	1	*Burkholderia cenocepacia*	GU433447	100
S2	IBa219 [AB904953]	621	*Burkholderia*	1	*Burkholderia cenocepacia*	GU433447	100
S2	IBa220 [AB904954]	619	*Burkholderia*	1	*Burkholderia cenocepacia*	GU433447	99.84
S2	IBa221 [AB904955]	616	*Burkholderia*	0.98	*Burkholderia cenocepacia*	FJ870663	98.7
S2	IBa222 [AB904956]	622	*Burkholderia*	1	*Burkholderia cenocepacia*	GU433447	100
S2	IBa223 [AB904957]	622	*Burkholderia*	1	*Burkholderia cenocepacia*	GU433447	100
S2	IBa224 [AB904958]	609	*Burkholderia*	1	*Burkholderia* sp.	FJ560464	99.84
S2	IBa225 [AB904959]	623	*Burkholderia*	1	*Burkholderia cenocepacia*	GU433447	100
S2	IBa226 [AB904960]	622	*Burkholderia*	1	*Burkholderia* sp.	FJ560464	99.84
S2	IBa227 [AB904961]	616	*Burkholderia*	1	*Burkholderia* sp.	FJ560464	99.84
S2	IBa228 [AB904962]	629	*Burkholderia*	1	*Burkholderia cenocepacia*	GU433447	99.84
S2	IBa229 [AB904963]	632	*Burkholderia*	1	*Burkholderia cenocepacia*	GU433447	100
S2	IBa230 [AB904964]	626	*Burkholderia*	1	*Burkholderia cenocepacia*	GU433447	99.68

S2	IBa231 [AB904965]	631	*Burkholderia*	1	*Burkholderia cenocepacia*	GU433447	100
S2	IBa232 [AB904966]	631	*Burkholderia*	1	*Burkholderia cenocepacia*	GU433447	100
S2	IBa233 [AB904967]	624	*Burkholderia*	1	*Burkholderia cenocepacia*	GU433447	100
S2	IBa234 [AB904968]	619	*Burkholderia*	1	*Burkholderia cenocepacia*	FJ870663	99.84
S2	IBa235 [AB904969]	600	*Burkholderia*	0.99	*Burkholderia* sp.	FJ560464	98.83
S2	IBa236 [AB904970]	626	*Burkholderia*	0.99	*Burkholderia* sp.	AB508895	99.84
S2	IBa237 [AB904971]	632	*Burkholderia*	1	*Burkholderia cenocepacia*	GU433447	100
S2	IBa238 [AB904972]	632	*Burkholderia*	1	*Burkholderia cenocepacia*	GU433447	100
S2	IBa239 [AB904973]	614	*Burkholderia*	1	*Burkholderia* sp.	FJ560464	99.84
S2	IBa240 [AB904974]	626	*Burkholderia*	1	*Burkholderia caribensis*	HM582870	99.68
S2	IBa241 [AB904975]	622	*Burkholderia*	1	*Burkholderia cenocepacia*	GU433447	100
S2	IBa242 [AB904976]	627	*Burkholderia*	1	*Burkholderia cenocepacia*	GU433447	100
S2	IBa243 [AB904977]	626	*Burkholderia*	1	*Burkholderia cenocepacia*	GU433447	100
S2	IBa244 [AB904978]	632	*Burkholderia*	1	*Burkholderia cenocepacia*	GU433447	100
S2	IBa245 [AB904979]	620	*Burkholderia*	0.99	*Burkholderia cenocepacia*	GU433447	99.84
S2	IBa246 [AB904980]	617	*Burkholderia*	1	*Burkholderia* sp.	FJ560464	100
S2	IBa247 [AB904981]	624	*Burkholderia*	1	*Burkholderia* sp.	AB508895	99.84
S2	IBa248 [AB904982]	620	*Burkholderia*	1	*Burkholderia* sp.	GU292560	99.52
S2	IBa249 [AB904983]	626	*Burkholderia*	1	*Burkholderia* sp.	FJ560464	100
S2	IBa250 [AB904984]	630	*Burkholderia*	1	*Burkholderia cenocepacia*	GU433447	99.84
S2	IBa251 [AB904985]	622	*Burkholderia*	1	*Burkholderia cenocepacia*	GU433447	100
S2	IBa252 [AB904986]	630	*Burkholderia*	1	*Burkholderia cenocepacia*	GU433447	100
S2	IBa253 [AB904987]	626	*Burkholderia*	1	*Burkholderia cenocepacia*	GU433447	100
S2	IBa254 [AB904988]	629	*Burkholderia*	1	*Burkholderia cenocepacia*	GU433447	100
S2	IBa255 [AB904989]	626	*Burkholderia*	1	*Burkholderia cenocepacia*	GU433447	100
S2	IBa256 [AB904990]	618	*Burkholderia*	0.99	*Burkholderia cenocepacia*	FJ870663	99.19
S2	IBa257 [AB904991]	626	*Burkholderia*	1	*Burkholderia cenocepacia*	GU433447	99.84
S2	IBa258 [AB904992]	627	*Burkholderia*	0.84	*Burkholderia cenocepacia*	FJ870663	97.45
S2	IBa259 [AB904993]	614	*Burkholderia*	1	*Burkholderia* sp.	FJ560464	99.67
S2	IBa260 [AB904994]	620	*Burkholderia*	1	*Burkholderia cenocepacia*	GU433447	100
S2	IBa261 [AB904995]	621	*Burkholderia*	1	*Burkholderia* sp.	FJ560464	100
S2	IBa262 [AB904996]	622	*Burkholderia*	1	*Burkholderia* sp.	FJ560464	99.84
S2	IBa263 [AB904997]	630	*Burkholderia*	1	*Burkholderia cenocepacia*	GU433447	100
S2	IBa264 [AB904998]	627	*Burkholderia*	1	*Burkholderia cenocepacia*	GU433447	100
S2	IBa265 [AB904999]	624	*Burkholderia*	1	*Burkholderia cenocepacia*	GU433447	100
S2	IBa266 [AB905000]	623	*Burkholderia*	1	*Burkholderia cenocepacia*	GU433447	100

**S2**	**IBa267 [AB905001]**	**629**	***Burkholderia***	**1**	***Burkholderia cenocepacia***	**GU433447**	**100**
**S2**	**IBa268 [AB905002]**	**618**	***Burkholderia***	**1**	***Burkholderia cenocepacia***	**GU433447**	**100**
**S2**	**IBa269 [AB905003]**	**626**	***Burkholderia***	**1**	***Burkholderia cenocepacia***	**FJ870663**	**99.84**
**S2**	**IBa270 [AB905004]**	**619**	***Burkholderia***	**1**	***Burkholderia cenocepacia***	**CP000460**	**99.68**
**S2**	**IBa271 [AB905005]**	**615**	***Burkholderia***	**1**	***Burkholderia cenocepacia***	**FJ870663**	**99.84**
**S2**	**IBa272 [AB905006]**	**623**	***Burkholderia***	**1**	***Burkholderia cenocepacia***	**GU433447**	**100**
**S2**	**IBa273 [AB905007]**	**623**	***Burkholderia***	**0.99**	***Burkholderia cenocepacia***	**FJ870663**	**98.39**
**S2**	**IBa274 [AB905008]**	**619**	***Burkholderia***	**1**	***Burkholderia cenocepacia***	**GU433447**	**100**
**S2**	**IBa275 [AB905009]**	**631**	***Burkholderia***	**1**	***Burkholderia cenocepacia***	**GU433447**	**100**
**S2**	**IBa276 [AB905010]**	**621**	***Burkholderia***	**1**	***Burkholderia cenocepacia***	**GU433447**	**99.68**
**S2**	**IBa277 [AB905011]**	**620**	***Burkholderia***	**1**	***Burkholderia cenocepacia***	**GU433447**	**99.52**
**S2**	**IBa278 [AB905012]**	**626**	***Burkholderia***	**1**	***Burkholderia cenocepacia***	**GU433447**	**100**
**S2**	**IBa279 [AB905013]**	**632**	***Burkholderia***	**1**	***Burkholderia cenocepacia***	**GU433447**	**100**
**S2**	**IBa280 [AB905014]**	**617**	***Burkholderia***	**1**	***Burkholderia* sp.**	**FJ560464**	**100**
**S2**	**IBa281 [AB905015]**	**630**	***Burkholderia***	**1**	***Burkholderia cenocepacia***	**GU433447**	**100**
**S2**	**IBa282 [AB905016]**	**622**	***Burkholderia***	**1**	***Burkholderia cenocepacia***	**GU433447**	**100**
**S2**	**IBa283 [AB905017]**	**613**	***Burkholderia***	**1**	***Burkholderia* sp.**	**FJ560464**	**99.84**
**S2**	**IBa284 [AB905018]**	**631**	***Burkholderia***	**1**	***Burkholderia cenocepacia***	**GU433447**	**100**
**S2**	**IBa285 [AB905019]**	**615**	***Burkholderia***	**1**	***Burkholderia cenocepacia***	**FJ870663**	**100**
**S2**	**IBa286 [AB905020]**	**612**	***Burkholderia***	**1**	***Burkholderia* sp.**	**FJ560464**	**99.67**
**S2**	**IBa287 [AB905021]**	**616**	***Burkholderia***	**0.54**	***Burkholderia cenocepacia***	**FJ870663**	**97.08**
**S2**	**IBa288 [AB905022]**	**621**	***Burkholderia***	**1**	***Burkholderia cenocepacia***	**GU433447**	**100**
**S2**	**IBa289 [AB905023]**	**618**	***Burkholderia***	**1**	***Burkholderia* sp.**	**FJ560464**	**99.84**
**S2**	**IBa290 [AB905024]**	**600**	***Burkholderia***	**0.97**	***Burkholderia* sp.**	**FJ560464**	**97.83**
**S2**	**IBa291 [AB905025]**	**616**	***Burkholderia***	**1**	***Burkholderia* sp.**	**FJ560464**	**100**
**S2**	**IBa292 [AB905026]**	**616**	***Burkholderia***	**1**	***Burkholderia* sp.**	**FJ560464**	**100**
**S2**	**IBa293 [AB905027]**	**621**	***Burkholderia***	**1**	***Burkholderia cenocepacia***	**GU433447**	**100**
**S2**	**IBa294 [AB905028]**	**626**	***Burkholderia***	**1**	***Burkholderia* sp.**	**FJ560464**	**100**
**S3**	**IBa301 [AB905029]**	**633**	***Burkholderia***	**1**	***Burkholderia cenocepacia***	**GU433447**	**100**
**S3**	**IBa302 [AB905030]**	**633**	***Burkholderia***	**1**	***Burkholderia cenocepacia***	**GU433447**	**100**
**S3**	**IBa303 [AB905031]**	**633**	***Burkholderia***	**1**	***Burkholderia cenocepacia***	**GU433447**	**100**
**S3**	**IBa304 [AB905032]**	**638**	***Burkholderia***	**1**	***Burkholderia cenocepacia***	**GU433447**	**99.84**
**S3**	**IBa305 [AB905033]**	**631**	***Burkholderia***	**1**	***Burkholderia cenocepacia***	**GU433447**	**100**
**S3**	**IBa306 [AB905034]**	**619**	***Burkholderia***	**1**	***Burkholderia cenocepacia***	**FJ870663**	**99.68**
**S3**	**IBa307 [AB905035]**	**632**	***Burkholderia***	**1**	***Burkholderia cenocepacia***	**GU433447**	**100**
**S3**	**IBa308 [AB905036]**	**633**	***Burkholderia***	**1**	***Burkholderia cenocepacia***	**GU433447**	**100**

**S3**	**IBa309 [AB905037]**	**633**	***Burkholderia***	**1**	***Burkholderia cenocepacia***	**GU433447**	**100**
**S3**	**IBa310 [AB905038]**	**632**	***Burkholderia***	**1**	***Burkholderia cenocepacia***	**GU433447**	**100**
**S3**	**IBa311 [AB905039]**	**632**	***Burkholderia***	**1**	***Burkholderia cenocepacia***	**GU433447**	**100**
**S3**	**IBa312 [AB905040]**	**628**	***Burkholderia***	**1**	***Burkholderia vietnamiensis***	**AB568311**	**99.68**
**S3**	**IBa313 [AB905041]**	**632**	***Burkholderia***	**1**	***Burkholderia cenocepacia***	**GU433447**	**100**
**S3**	**IBa314 [AB905042]**	**632**	***Burkholderia***	**1**	***Burkholderia cenocepacia***	**GU433447**	**100**
**S3**	**IBa315 [AB905043]**	**624**	***Burkholderia***	**1**	***Burkholderia caribensis***	**HM582870**	**99.68**
**S3**	**IBa316 [AB905044]**	**632**	***Burkholderia***	**1**	***Burkholderia cenocepacia***	**GU433447**	**100**
**S3**	**IBa317 [AB905045]**	**632**	***Burkholderia***	**1**	***Burkholderia cenocepacia***	**GU433447**	**100**
**S3**	**IBa318 [AB905046]**	**633**	***Burkholderia***	**1**	***Burkholderia cenocepacia***	**GU433447**	**100**
**S3**	**IBa319 [AB905047]**	**633**	***Burkholderia***	**1**	***Burkholderia cenocepacia***	**GU433447**	**100**
**S3**	**IBa320 [AB905048]**	**633**	***Burkholderia***	**1**	***Burkholderia cenocepacia***	**GU433447**	**100**
**S3**	**IBa321 [AB905049]**	**630**	***Burkholderia***	**1**	***Burkholderia cenocepacia***	**GU433447**	**100**
**S3**	**IBa322 [AB905050]**	**632**	***Burkholderia***	**1**	***Burkholderia cenocepacia***	**GU433447**	**100**
**S3**	**IBa323 [AB905051]**	**632**	***Burkholderia***	**1**	***Burkholderia cenocepacia***	**GU433447**	**100**
**S3**	**IBa324 [AB905052]**	**633**	***Burkholderia***	**1**	***Burkholderia cenocepacia***	**GU433447**	**100**
**S3**	**IBa325 [AB905053]**	**632**	***Burkholderia***	**1**	***Burkholderia cenocepacia***	**GU433447**	**100**
**S3**	**IBa326 [AB905054]**	**633**	***Burkholderia***	**1**	***Burkholderia cenocepacia***	**GU433447**	**100**
**S3**	**IBa327 [AB905055]**	**633**	***Burkholderia***	**1**	***Burkholderia cenocepacia***	**GU433447**	**100**
**S3**	**IBa328 [AB905056]**	**632**	***Burkholderia***	**1**	***Burkholderia cenocepacia***	**GU433447**	**100**
**S3**	**IBa329 [AB905057]**	**632**	***Burkholderia***	**1**	***Burkholderia cenocepacia***	**GU433447**	**100**
**S3**	**IBa330 [AB905058]**	**633**	***Burkholderia***	**1**	***Burkholderia cenocepacia***	**GU433447**	**100**
**S3**	**IBa331 [AB905059]**	**631**	***Burkholderia***	**1**	***Burkholderia cenocepacia***	**GU433447**	**100**
**S3**	**IBa332 [AB905060]**	**630**	***Burkholderia***	**1**	***Burkholderia cenocepacia***	**GU433447**	**100**
**S3**	**IBa333 [AB905061]**	**631**	***Burkholderia***	**1**	***Burkholderia cenocepacia***	**GU433447**	**100**
**S3**	**IBa334 [AB905062]**	**631**	***Burkholderia***	**1**	***Burkholderia cenocepacia***	**GU433447**	**100**
**S3**	**IBa335 [AB905063]**	**638**	***Burkholderia***	**1**	***Burkholderia cenocepacia***	**GU433447**	**99.84**
**S3**	**IBa336 [AB905064]**	**629**	***Burkholderia***	**1**	***Burkholderia* sp.**	**FJ560464**	**99.84**
**S3**	**IBa337 [AB905065]**	**627**	***Burkholderia***	**1**	***Burkholderia cenocepacia***	**GU433447**	**100**
**S3**	**IBa338 [AB905066]**	**633**	***Burkholderia***	**1**	***Burkholderia cenocepacia***	**GU433447**	**99.84**
**S3**	**IBa339 [AB905067]**	**632**	***Burkholderia***	**1**	***Burkholderia cenocepacia***	**GU433447**	**100**
**S3**	**IBa340 [AB905068]**	**633**	***Burkholderia***	**1**	***Burkholderia cenocepacia***	**GU433447**	**100**
**S3**	**IBa341 [AB905069]**	**632**	***Burkholderia***	**1**	***Burkholderia cenocepacia***	**GU433447**	**100**
**S3**	**IBa342 [AB905070]**	**633**	***Burkholderia***	**1**	***Burkholderia cenocepacia***	**GU433447**	**100**
**S3**	**IBa343 [AB905071]**	**631**	***Burkholderia***	**1**	***Burkholderia cenocepacia***	**GU433447**	**100**
**S3**	**IBa344 [AB905072]**	**633**	***Burkholderia***	**1**	***Burkholderia cenocepacia***	**GU433447**	**100**

**S3**	**IBa345 [AB905073]**	**632**	***Burkholderia***	**1**	***Burkholderia cenocepacia***	**GU433447**	**100**
**S3**	**IBa346 [AB905074]**	**633**	***Burkholderia***	**1**	***Burkholderia cenocepacia***	**GU433447**	**100**
**S3**	**IBa347 [AB905075]**	**633**	***Burkholderia***	**1**	***Burkholderia cenocepacia***	**GU433447**	**100**
**S3**	**IBa348 [AB905076]**	**632**	***Burkholderia***	**1**	***Burkholderia cenocepacia***	**GU433447**	**100**
**S3**	**IBa349 [AB905077]**	**633**	***Burkholderia***	**1**	***Burkholderia cenocepacia***	**GU433447**	**100**
**S3**	**IBa350 [AB905078]**	**633**	***Burkholderia***	**1**	***Burkholderia cenocepacia***	**GU433447**	**100**
**S3**	**IBa351 [AB905079]**	**633**	***Burkholderia***	**1**	***Burkholderia cenocepacia***	**GU433447**	**100**
**S3**	**IBa352 [AB905080]**	**630**	***Burkholderia***	**1**	***Burkholderia cenocepacia***	**GU433447**	**100**
**S3**	**IBa353 [AB905081]**	**632**	***Burkholderia***	**1**	***Burkholderia cenocepacia***	**GU433447**	**100**
**S3**	**IBa354 [AB905082]**	**632**	***Burkholderia***	**1**	***Burkholderia cenocepacia***	**GU433447**	**100**
**S3**	**IBa355 [AB905083]**	**633**	***Burkholderia***	**1**	***Burkholderia cenocepacia***	**GU433447**	**100**
**S3**	**IBa356 [AB905084]**	**633**	***Burkholderia***	**1**	***Burkholderia cenocepacia***	**GU433447**	**100**
**S3**	**IBa357 [AB905085]**	**633**	***Burkholderia***	**1**	***Burkholderia cenocepacia***	**GU433447**	**100**
**S3**	**IBa358 [AB905086]**	**632**	***Burkholderia***	**1**	***Burkholderia cenocepacia***	**GU433447**	**100**
**S3**	**IBa359 [AB905087]**	**635**	***Burkholderia***	**1**	***Burkholderia cenocepacia***	**GU433447**	**100**
**S3**	**IBa360 [AB905088]**	**632**	***Burkholderia***	**1**	***Burkholderia cenocepacia***	**GU433447**	**100**
**S3**	**IBa361 [AB905089]**	**632**	***Burkholderia***	**1**	***Burkholderia cenocepacia***	**GU433447**	**100**
**S3**	**IBa362 [AB905090]**	**632**	***Burkholderia***	**1**	***Burkholderia cenocepacia***	**GU433447**	**100**
**S3**	**IBa363 [AB905091]**	**632**	***Burkholderia***	**1**	***Burkholderia cenocepacia***	**GU433447**	**100**
**S3**	**IBa364 [AB905092]**	**631**	***Burkholderia***	**1**	***Burkholderia cenocepacia***	**GU433447**	**100**
**S3**	**IBa365 [AB905093]**	**632**	***Burkholderia***	**1**	***Burkholderia cenocepacia***	**GU433447**	**100**
**S3**	**IBa366 [AB905094]**	**632**	***Burkholderia***	**1**	***Burkholderia cenocepacia***	**GU433447**	**100**
**S3**	**IBa367 [AB905095]**	**632**	***Burkholderia***	**1**	***Burkholderia cenocepacia***	**GU433447**	**100**
**S3**	**IBa368 [AB905096]**	**633**	***Burkholderia***	**1**	***Burkholderia cenocepacia***	**GU433447**	**100**
**S3**	**IBa369 [AB905097]**	**632**	***Burkholderia***	**1**	***Burkholderia cenocepacia***	**GU433447**	**100**
**S3**	**IBa370 [AB905098]**	**633**	***Burkholderia***	**1**	***Burkholderia caribensis***	**HM582870**	**99.68**
**S3**	**IBa371 [AB905099]**	**632**	***Burkholderia***	**1**	***Burkholderia cenocepacia***	**GU433447**	**100**
**S3**	**IBa372 [AB905100]**	**633**	***Burkholderia***	**1**	***Burkholderia cenocepacia***	**GU433447**	**100**
**S3**	**IBa373 [AB905101]**	**616**	***Pandoraea***	**1**	***Pandoraea pulmonicola***	**NR028750**	**99.68**
**S3**	**IBa374 [AB905102]**	**632**	***Burkholderia***	**1**	***Burkholderia cenocepacia***	**GU433447**	**100**
**S3**	**IBa375 [AB905103]**	**633**	***Burkholderia***	**1**	***Burkholderia cenocepacia***	**GU433447**	**100**
**S3**	**IBa376 [AB905104]**	**633**	***Burkholderia***	**1**	***Burkholderia cenocepacia***	**GU433447**	**100**
**S3**	**IBa377 [AB905105]**	**632**	***Burkholderia***	**1**	***Burkholderia cenocepacia***	**GU433447**	**100**
**S3**	**IBa378 [AB905106]**	**632**	***Burkholderia***	**1**	***Burkholderia cenocepacia***	**GU433447**	**100**
**S3**	**IBa379 [AB905107]**	**632**	***Burkholderia***	**1**	***Burkholderia cenocepacia***	**GU433447**	**100**
**S3**	**IBa380 [AB905108]**	**633**	***Burkholderia***	**1**	***Burkholderia cenocepacia***	**GU433447**	**100**

**S3**	**IBa381 [AB905109]**	**627**	***Burkholderia***	**1**	***Burkholderia cenocepacia***	**GU433447**	**100**
**S3**	**IBa382 [AB905110]**	**633**	***Pandoraea***	**0.6**	***Pandoraea pulmonicola***	**NR028750**	**99.68**
**S3**	**IBa383 [AB905111]**	**633**	***Burkholderia***	**1**	***Burkholderia cenocepacia***	**GU433447**	**100**
**S3**	**IBa384 [AB905112]**	**633**	***Burkholderia***	**1**	***Burkholderia cenocepacia***	**GU433447**	**100**
**S3**	**IBa385 [AB905113]**	**632**	***Burkholderia***	**1**	***Burkholderia cenocepacia***	**GU433447**	**100**
**S3**	**IBa386 [AB905114]**	**632**	***Burkholderia***	**1**	***Burkholderia cenocepacia***	**GU433447**	**100**
**S3**	**IBa387 [AB905115]**	**632**	***Pandoraea***	**0.94**	***Pandoraea* sp.**	**HM125147**	**99.68**
**S3**	**IBa388 [AB905116]**	**633**	***Burkholderia***	**1**	***Burkholderia cenocepacia***	**GU433447**	**100**
**S3**	**IBa389 [AB905117]**	**633**	***Burkholderia***	**1**	***Burkholderia cenocepacia***	**GU433447**	**100**
**S3**	**IBa390 [AB905118]**	**632**	***Burkholderia***	**1**	***Burkholderia cenocepacia***	**GU433447**	**100**
**S3**	**IBa391 [AB905119]**	**633**	***Burkholderia***	**1**	***Burkholderia cenocepacia***	**GU433447**	**100**
**S3**	**IBa392 [AB905120]**	**632**	***Burkholderia***	**1**	***Burkholderia cenocepacia***	**GU433447**	**100**
**S3**	**IBa393 [AB905121]**	**633**	***Burkholderia***	**1**	***Burkholderia cenocepacia***	**GU433447**	**100**
**S3**	**IBa394 [AB905122]**	**632**	***Burkholderia***	**1**	***Burkholderia cenocepacia***	**GU433447**	**100**
**S3**	**IBa395 [AB905123]**	**604**	***Burkholderia***	**0.85**	***Burkholderia* sp.**	**FJ560464**	**98.34**
**S3**	**IBa396 [AB905124]**	**630**	***Burkholderia***	**1**	***Burkholderia cenocepacia***	**GU433447**	**100**
**N3**	**IBb301 [AB905125]**	**611**	***Achromobacter***	**0.94**	***Achromobacter* sp.**	**GU244494**	**99.51**
**N3**	**IBb302 [AB905126]**	**611**	***Dyella***	**0.9**	***Dyella* sp.**	**AB366319**	**97.05**
**N3**	**IBb303 [AB905127]**	**615**	***Dyella***	**1**	***Dyella* sp.**	**AB366319**	**99.84**
**N3**	**IBb304 [AB905128]**	**634**	***Burkholderia***	**1**	***Burkholderia* sp.**	**AB208548**	**100**
**N3**	**IBb305 [AB905129]**	**633**	***Burkholderia***	**1**	***Burkholderia* sp.**	**AB208548**	**100**
**N3**	**IBb306 [AB905130]**	**630**	***Dyella***	**1**	***Dyella* sp.**	**AB366319**	**100**
**N3**	**IBb307 [AB905131]**	**628**	***Dyella***	**1**	***Dyella* sp.**	**AB366319**	**100**
**N3**	**IBb308 [AB905132]**	**632**	***Dyella***	**1**	***Dyella* sp.**	**AB366319**	**97.63**
**N3**	**IBb309 [AB905133]**	**633**	***Burkholderia***	**0.99**	***Burkholderia* sp.**	**AB208548**	**100**
**N3**	**IBb310 [AB905134]**	**634**	***Burkholderia***	**1**	***Burkholderia* sp.**	**AB208548**	**100**
**N3**	**IBb311 [AB905135]**	**629**	***Burkholderia***	**0.98**	***Burkholderia* sp.**	**AB508895**	**99.21**
**N3**	**IBb312 [AB905136]**	**634**	***Burkholderia***	**1**	***Burkholderia* sp.**	**AB208548**	**100**
**N3**	**IBb313 [AB905137]**	**629**	***Dyella***	**1**	***Dyella* sp.**	**AB366319**	**100**
**N3**	**IBb314 [AB905138]**	**608**	***Dyella***	**0.98**	***Dyella* sp.**	**AB366319**	**97.2**
**N3**	**IBb315 [AB905139]**	**622**	***Dyella***	**1**	***Dyella* sp.**	**AB366319**	**100**
**N3**	**IBb316 [AB905140]**	**612**	***Dyella***	**1**	***Dyella* sp.**	**AB366319**	**99.18**
**N3**	**IBb317 [AB905141]**	**633**	***Burkholderia***	**1**	***Burkholderia* sp.**	**AB508895**	**100**
**N3**	**IBb318 [AB905142]**	**627**	***Dyella***	**1**	***Dyella* sp.**	**AB366319**	**100**
**N3**	**IBb319 [AB905143]**	**632**	***Burkholderia***	**1**	***Burkholderia* sp.**	**AB208548**	**100**
**N3**	**IBb320 [AB905144]**	**614**	***Ralstonia***	**1**	***Ralstonia* sp.**	**GU936705**	**99.19**

**N3**	**IBb321 [AB905145]**	**628**	***Dyella***	**1**	***Dyella* sp.**	**AB366319**	**99.84**
**N3**	**IBb322 [AB905146]**	**638**	***Burkholderia***	**1**	***Burkholderia* sp.**	**AB208548**	**99.84**
**N3**	**IBb323 [AB905147]**	**608**	***Burkholderia***	**0.55**	***Burkholderia* sp.**	**AB208548**	**98.68**
**N3**	**IBb324 [AB905148]**	**631**	***Cupriavidus***	**1**	***Cupriavidus* sp.**	**EU827498**	**100**
**N3**	**IBb325 [AB905149]**	**631**	***Dyella***	**1**	***Dyella* sp.**	**AB366319**	**100**
**N3**	**IBb326 [AB905150]**	**628**	***Dyella***	**1**	***Dyella* sp.**	**AB366319**	**100**
**N3**	**IBb327 [AB905151]**	**640**	***Burkholderia***	**0.98**	***Burkholderia* sp.**	**AB208548**	**99.84**
**N3**	**IBb328 [AB905152]**	**634**	***Burkholderia***	**0.95**	***Burkholderia* sp.**	**AB232337**	**100**
**N3**	**IBb329 [AB905153]**	**631**	***Pandoraea***	**0.99**	***Pandoraea sputorum***	**AB480701**	**100**
**N3**	**IBb330 [AB905154]**	**632**	***Burkholderia***	**1**	***Burkholderia* sp.**	**AB208548**	**100**
**N3**	**IBb331 [AB905155]**	**640**	***Burkholderia***	**0.95**	***Burkholderia* sp.**	**AB232337**	**99.84**
**N3**	**IBb332 [AB905156]**	**633**	***Burkholderia***	**1**	***Burkholderia* sp.**	**AB508895**	**100**
**N3**	**IBb333 [AB905157]**	**633**	***Dyella***	**1**	***Dyella* sp.**	**AB366319**	**100**
**N3**	**IBb334 [AB905158]**	**641**	***Burkholderia***	**0.96**	***Burkholderia* sp.**	**AB232337**	**99.69**
**N3**	**IBb335 [AB905159]**	**632**	***Burkholderia***	**0.99**	***Burkholderia* sp.**	**AB208548**	**100**
**N3**	**IBb336 [AB905160]**	**628**	***Dyella***	**1**	***Dyella* sp.**	**AB366319**	**99.52**
**N3**	**IBb337 [AB905161]**	**622**	***Burkholderia***	**1**	***Burkholderia cenocepacia***	**GU433447**	**100**
**N3**	**IBb338 [AB905162]**	**631**	***Achromobacter***	**0.95**	***Achromobacter sp.***	**FJ665505**	**96.2**
**N3**	**IBb339 [AB905163]**	**630**	***Burkholderia***	**1**	***Burkholderia* sp.**	**AB232337**	**99.84**
**N3**	**IBb340 [AB905164]**	**634**	***Burkholderia***	**1**	***Burkholderia* sp.**	**HM107185**	**100**
**N3**	**IBb341 [AB905165]**	**633**	***Burkholderia***	**0.96**	***Burkholderia* sp.**	**AB208548**	**100**
**N3**	**IBb342 [AB905166]**	**628**	***Dyella***	**1**	***Dyella* sp.**	**AB366319**	**98.73**
**N3**	**IBb343 [AB905167]**	**634**	***Burkholderia***	**0.97**	***Burkholderia* sp.**	**AB232337**	**99.84**
**N3**	**IBb344 [AB905168]**	**619**	***Burkholderia***	**0.99**	***Burkholderia* sp.**	**AM992535**	**99.35**
**N3**	**IBb345 [AB905169]**	**619**	***Dyella***	**1**	***Dyella* sp.**	**AB366319**	**99.68**
**N3**	**IBb346 [AB905170]**	**634**	***Burkholderia***	**1**	***Burkholderia* sp.**	**AB208548**	**100**
**N3**	**IBb347 [AB905171]**	**630**	***Dyella***	**1**	***Dyella* sp.**	**AB366319**	**99.84**
**N3**	**IBb348 [AB905172]**	**634**	***Burkholderia***	**0.98**	***Burkholderia* sp.**	**AB232337**	**99.84**
**N3**	**IBb349 [AB905173]**	**619**	***Burkholderia***	**0.98**	***Burkholderia* sp.**	**GU292560**	**99.19**
**N3**	**IBb350 [AB905174]**	**634**	***Burkholderia***	**1**	***Burkholderia* sp.**	**AB208548**	**100**
**N3**	**IBb351 [AB905175]**	**633**	***Burkholderia***	**0.99**	***Burkholderia* sp.**	**AB232337**	**100**
**N3**	**IBb352 [AB905176]**	**614**	***Dyella***	**1**	***Dyella* sp.**	**AB366319**	**99.02**
**N3**	**IBb353 [AB905177]**	**633**	***Burkholderia***	**0.98**	***Burkholderia* sp.**	**AB232337**	**100**
**N3**	**IBb354 [AB905178]**	**620**	***Pandoraea***	**1**	***Pandoraea norimbergensis***	**AY268174**	**100**
**N3**	**IBb355 [AB905179]**	**639**	***Burkholderia***	**0.99**	***Burkholderia* sp.**	**AB208548**	**99.84**
**N3**	**IBb356 [AB905180]**	**632**	***Burkholderia***	**1**	***Burkholderia* sp.**	**AB208548**	**100**

**N3**	**IBb357 [AB905181]**	**633**	***Burkholderia***	**1**	***Burkholderia* sp.**	**AB208548**	**100**
**N3**	**IBb358 [AB905182]**	**625**	***Dyella***	**1**	***Dyella* sp.**	**AB366319**	**99.36**
**N3**	**IBb359 [AB905183]**	**640**	***Burkholderia***	**1**	***Burkholderia* sp.**	**AB208548**	**99.84**
**N3**	**IBb360 [AB905184]**	**627**	***Burkholderia***	**0.99**	***Burkholderia* sp.**	**AB208548**	**100**
**N3**	**IBb361 [AB905185]**	**631**	***Burkholderia***	**1**	***Burkholderia* sp.**	**GU573900**	**99.52**
**N3**	**IBb362 [AB905186]**	**611**	***Burkholderia***	**0.93**	***Burkholderia* sp.**	**AB208548**	**99.51**
**N3**	**IBb363 [AB905187]**	**633**	***Burkholderia***	**0.98**	***Burkholderia* sp.**	**AB232337**	**100**
**N3**	**IBb364 [AB905188]**	**638**	***Burkholderia***	**1**	***Burkholderia* sp.**	**AB208548**	**99.84**
**N3**	**IBb365 [AB905189]**	**627**	***Burkholderia***	**0.98**	***Burkholderia* sp.**	**AB208548**	**100**
**N3**	**IBb366 [AB905190]**	**640**	***Burkholderia***	**0.99**	***Burkholderia* sp.**	**AB208548**	**99.84**
**N3**	**IBb367 [AB905191]**	**633**	***Burkholderia***	**0.99**	***Burkholderia* sp.**	**AB208548**	**100**
**N3**	**IBb368 [AB905192]**	**620**	***Burkholderia***	**0.99**	***Burkholderia* sp.**	**HM101281**	**99.03**
**N3**	**IBb369 [AB905193]**	**622**	***Dyella***	**1**	***Dyella* sp.**	**AB366319**	**100**
**N3**	**IBb370 [AB905194]**	**634**	***Burkholderia***	**0.98**	***Burkholderia* sp.**	**AB208548**	**100**
**N3**	**IBb371 [AB905195]**	**639**	***Burkholderia***	**1**	***Burkholderia* sp.**	**HM107185**	**99.84**
**N3**	**IBb372 [AB905196]**	**632**	***Burkholderia***	**0.98**	***Burkholderia* sp.**	**AB232337**	**99.84**

[a] Isolated from soil S after the 2nd and 3rd MEP-treatments (S2 and S3), and soil N after the 3rd MEP-treatment (N3).
